# Systematic Studies on Surface Erosion of Photocrosslinked Polyanhydride Tablets and Data Correlation with Release Kinetic Models

**DOI:** 10.3390/polym12051105

**Published:** 2020-05-12

**Authors:** Armin Geraili, Kibret Mequanint

**Affiliations:** 1 School of Biomedical Engineering, The University of Western Ontario, London, ON N6A 5B9, Canada; ageraili@uwo.ca; 2 Department of Chemical and Biochemical Engineering, The University of Western Ontario, London, ON N6A 5B9, Canada

**Keywords:** photocrosslinked polyanhydrides, erosion kinetics, surface erosion, mass loss, controlled-release drug delivery systems

## Abstract

Photocrosslinkable polyanhydrides that undergo surface erosion are suitable materials for controlled-release drug delivery systems. Investigating the impact of different parameters on their erosion behavior is essential before use in drug delivery systems. Although their synthesis is well-established, parameters that may substantially affect the erosion of thiol-ene polyanhydrides including temperature and pH of the media, the geometry of the polymers, and the media shaking rate (the convective force for the polymer erosion), have not yet been studied. This study explores the effects of different environmental and geometric parameters on mass loss (erosion) profiles of polyanhydrides synthesized by thiol-ene photopolymerization. A comparative study on several release kinetic models fitting is also described for a better understanding of the polymer erosion behavior. The results demonstrated that although the temperature was the only parameter that affected the induction period substantially, the mass-loss rate was influenced by the polymer composition, tablet geometry, temperature, pH, and mass transfer (shaking) rate. With regard to geometrical parameters, polymers with the same surface area to volume ratios showed similar mass loss trends despite their various volumes and surface areas. The mass loss of polyanhydride tablets with more complicated geometries than a simple slab was shown to be non-linear, and the kinetic model study indicated the dominant surface erosion mechanism. The results of this study allow for designing and manufacturing efficient delivery systems with a high-predictable drug release required in precision medicine using surface-erodible polyanhydrides.

## 1. Introduction

Erosion is the mechanism governing a broad range of chemically mediated controlled-release systems (CRSs) and can occur in the form of bulk and/or surface erosion. Most of the biodegradable polymers used in CRSs, e.g., polyesters, undergo bulk erosion [1]. Bulk-erodible polymers lose their mass from their interior and exterior simultaneously, resulting in low predictability of the drug release kinetics from these polymers. In contrast, surface-erodible polymers lose their mass from their surface (akin ice cube melting), and therefore their initial geometry is preserved while the size decreases with time. Moreover, the physical, chemical, and mechanical properties of surface-erodible polymers do not change during the erosion since the remaining mass has the same molecular weight as the starting polymer [2,3]. Thus, the highly reproducible and predictable release kinetics of surface-erodible polymers make them desirable for fabricating controlled drug delivery systems (DDSs) [4].

Polyanhydrides (PAHs) predominantly undergo surface erosion because of the hydrophobic backbone and hydrolytically unstable anhydride bonds in their structure. In comparison to other hydrolytically degradable bonds such as amides and esters, anhydride bonds are more unstable and have a short average half-life leading to faster degradation time. PAHs prepared by melt-condensation showed a linear mass loss profile in a slab geometry resulting in a near zero-order drug release [3,5]. PAHs maintain their surface eroding behavior down to a thickness of 20-100 μm, which is the smallest value reported [6,7]. Unlike other surface-erodible polymers such as polyorthoesters that undergo surface erosion only in acidic environments, PAHs have been shown to undergo surface erosion in acidic, alkaline, and neutral environments, albeit at different rates [8]. The most desired erosion-controlled DDS is the one that continues to release the loaded drugs until the erosion is completed. Depending on the target application, the erosion time can vary from a few hours to weeks or even years.

PAHs were studied for controlled DDSs in the early 1980s as an alternative to poly(D,L-lactic acid) (PLA), and poly(D,L-lactic-co-glycolic acid) (PLGA), which can only release drugs over few weeks [7,9]; however, the only FDA-approved drug delivery product from PAHs is the Gliadel^®^ Wafer (a polyanhydride-based implant for locally delivering cancer drug to brain tumors) [10]. One reason for their limited clinical use compared to other biodegradable polymers such as polyesters is due to the difficulties in synthesizing them [9]. The high reactivity of the anhydride functional groups in the vicinity of nucleophilic species such as water during polycondensation and the need to high temperature and low pressure poses challenges during synthesis [11]. As an alternative to ease the synthesis difficulty, photopolymerizable methacrylated anhydride monomers synthesized from dicarboxylic acids and methacrylic anhydride were investigated [12]. The benefit of photocrosslinkable polyanhydrides compared to those prepared by melt-condensation is their potential to be injectable for in situ crosslinking [13]. Further advances in photopolymerizable PAHs were prepared from commercially available 4-pentanoic anhydride (PNA) monomer and 2, 2-(ethylenedioxy) diethanethiol (3,6-dioxa-1,8-octane-dithiol) (EGDT), and pentaerythritol tetrakis(3-mercaptopropionate) (PETMP) crosslinkers via thiol-ene polymerization [11,14,15]. These recent developments expanded the library of photocrosslinkable polyanhydrides for biomedical applications. Not surprisingly, photocrosslinked PAHs prepared from PNA and PETMP also showed a linear mass loss profile, although non-linearity could be inferred for a cuboid geometry [16]. In addition, an induction period (lag time) of ~10 h is reported for thin (2 mm) slabs before surface erosion [14]. Advances made in synthesizing PAHs are not matched with a comprehensive erosion study as there are several parameters that can substantially affect the erosion of thiol-ene PAHs. For instance, if the controlled-release drug tablet is formulated for oral delivery, the appropriate geometry is a cylinder or a sphere, not a cuboid. Furthermore, interstitial fluid flow around the implant site or in the gastrointestinal (GI) tract affects the erosion kinetics. 

In this work, a systematic study on mass loss (surface erosion) of thiol-ene photopolymerized PAHs is presented, and the effects of various factors such as geometrical variables, media flow rate, temperature, and pH on mass loss are examined. The mass loss data indicated a clear non-linear behavior of the mass loss profiles. Moreover, for a better understanding of the erosion behavior of the PAHs tablets, release kinetic models were fit to the mass loss data using zero-order, first-order, Higuchi, Korsmeyer-Peppas, Hixon-Crowell, Hopfenberg, and Weibull kinetic models. Based on the best-fitted models, cylindrical and cuboid tablets obeyed the Hixson-Crowell and Hopfenberg models, which describe polymers with erosion mechanisms rather than diffusion-controlled systems. 

## 2. Experimental

### 2.1. Polymer Synthesis and Characterization

All monomers and the photoinitiator were purchased from Sigma-Aldrich (Milwaukee, WI) and were the highest purity available. The photocrosslinked polymers were synthesized from PNA, EGDT, and PETMP, using hydroxycyclohexyl phenyl ketone (HCPK) as a photoinitiator as reported elsewhere [14,15]. Briefly, HCPK (0.1 wt. % based on total monomer) was weighed and placed into a 15 mL Falcon tube. Then, PNA was transferred into the tube, followed by the mixture of EGDT and PETMP. The initial mole ratio of PNA to the total amount of thiol crosslinker groups (EGDT and PETMP) was 100:100. In this study, four different initial mole ratios of PNA to PETMP and EGDT were prepared and used: PNA: PETMP:EGDT = 100:100:0, 100:75:25, 100:50:50, and 100:25:75 [14]. The homogenous pre-polymer solution was exposed to ultraviolet (UV) light (CL-1000 UVP Cross-linker, Analytik Jena, Germany) equipped with 365 nm UV lamps (intensity:~5 mW/cm^2^) for 5 to 15 min. The synthesized polymers were characterized by Attenuated Total Reflectance Fourier-Transform Infrared Spectroscopy (ATR-FTIR, Vector 22 FTIR spectrometer, Bruker, Billerica, MA), Differential Scanning Calorimetry (DSC, Q20, TA instrument, New Castle, DE), Thermogravimetric analysis (TGA, Q50, TA instrument, New Castle, DE), water contact angle (WCA, DSA-100 Drop Shape Analyzer, Kruss, Hamburg, Germany), and x-ray diffraction (XRD, MiniFlex powder diffractometer, Rigaku, Tokyo, Japan). For some polymers, 1-3 wt. % Orange G (OG, Sigma Aldrich, Milwaukee, WI) as a model compound was added to the pre-polymer solution. 

### 2.2. Fabrication of 3D Printed Molds

Polydimethylsiloxane (PDMS) molds were fabricated using master molds printed by commercial DLP 3D printer (PICO2, Asiga, Sydney, Australia). Desired 3D models were designed by AutoCAD software and printed layer by layer from a UV-curable resin (Clear 2500T, Miicraft, Hsinchu, Taiwan). The layer thickness was set as 250 µm, and the irradiation time for each layer was 5 sec. The 3D printed objects were treated using sonication (Bransonic 3210-50/60 Hz, Branson, Danbury, CT) in ethanol for 10 min and washed by deionized (DI) water to remove any soluble fraction. Both the Sylgard 184 Silicone elastomer pre-polymer and the curing agent were purchased from Ellsworth Adhesive, ON, Canada, and were used for making PDMS.

### 2.3. Experimental Mass Loss Studies under Different Conditions 

The mass loss profiles of polymers with different initial mole ratios of PNA to PETMP and EGDT were studied. To obtain the mass loss profile, polymers were immersed in 5 mL phosphate-buffered saline (PBS, pH = 7.4) in glass vials, before being placed on a shaker (VWR micro-plate shaker). The shaker was set up at 37 °C with specific shaking rates (120 rpm). At certain time points (every hour), the polymers were extracted from the solution, the water on the surface of the polymer was carefully wiped off using the disposable wipes (Kimwipes), and the dry polymer was weighted using the weight balance. The parameters that were evaluated for mass loss profiles were the polymer composition, temperature, fluid flow around the samples (shaking), sample geometry, and pH.

To study the effect of polymer composition on the mass loss profile at room temperature and at physiological temperature (37 °C) cylindrical specimens from four different initial mole ratios of crosslinkers (PETMP:EGDT = 100:0, 75:25, 50:50, and 25:75) were used at pH = 7.4. To study the effect of pH, five PBS solutions with five different pH values (2.55, 4.27, 6.09, 7.41, and 7.98) were prepared to simulate the pH of different parts of GI tract. The mass loss measurements were conducted for polymer cubes (8.7 × 8.7 × 8.7 mm) consisting of PNA and PETMP monomers at room temperature. To investigate the effect of the mass transfer (mixing) on polymer mass loss profile, samples were placed at different shaking rates (0, 60, and 120 rpm). These experiments help us understand the impact of the velocity of the solution on mass loss of the polymer. To study the effect of dynamic conditions on mass loss, cylindrical specimens 8.7 mm × 8.7 mm (diameter × height) were placed in PBS at pH = 7.4 and shaken at 0, 60, and 120 rpm at 37 °C to mimic the GI tract environment. The polymers were removed from PBS and weighted every hour until the completion of the polymer mass loss experiments. The remaining mass percentage and the fractional mass loss percentage were calculated from:

(1) $$Fractional\;remaining\;mass\;percentage=\frac{M_{t}}{M_{0}}\times 100$$

(2) $$Fractional\;mass\;loss\;\left(mass\;eroded\right)\;percentage=\frac{M_{0}-{\;M}_{t}}{M_{0}}\times 100$$

where M_0_ is the initial mass of the polymer, and M_t_ is the polymer mass at time, t.

The effect of geometry on the mass loss profile was investigated by fabricating several cylindrical polymers with different diameters and heights. Firstly, small cylindrical shapes (3.3 mm diameter and 3.3 mm height) were fabricated from PAHs having crosslinker mole ratios of PETMP to EGDT of 100:0, and 75:25. The mass loss profile of the cylinders was then compared to bigger cylindrical shapes (diameter and height = 8.7 mm). Three separate experiments were designed to examine the effect of the polymer surface area, volume, and surface area to volume (SA/V) ratio on the mass loss profiles. In the first experiment, two cylindrical polymers with the same surface area were fabricated, with the volume of one sample 36 % higher than the other. In the second experiment, the volumes of the two cylinders were kept constant, and the surface areas had a 44 % difference. Finally, two cylindrical samples with different surface areas and volumes, but equal SA/V ratios were fabricated. 

### 2.4. Correlation of Erosion Data to Drug Release Kinetic Models 

The mass loss data were fitted to several kinetic models to determine the best fit and to gain insight into the mechanism of mass loss. The tested mass loss kinetic models are presented in Tables 2 and 3. 

## 3. Results and Discussion

### 3.1. Polymer Preparation and Characterization

We followed a reported procedure to synthesize these polymers [14]. ATR-FTIR results showed the absence of thiol and vinyl peaks at 2565 cm^–1^ and 1640-1650 cm^–1^, respectively, indicating that the thiol and vinyl functional groups were reacted as previously reported [15]. Furthermore, the dual peaks of anhydride functional groups were seen at 1730-1740 cm^–1^ (Supporting Figure S1). All the synthesized PAH polymers were amorphous (Supporting Figures S2 and S4). TGA studies demonstrated that (PNA:PETMP:EGDT = 100:100:0, 100:75:25, 100:50:50, 100:25:75) PAHs had thermal stability with the onset of decomposition temperature observed at 329 °C, 328 °C, 324 °C, and 317 °C, respectively (Supporting Figure S3). The different PAH polymers also had glass transition temperatures (Tg) between –25.1°C and –55.8°C depending on the crosslinker ratio. Increasing the ratio of tetra-thiol monomer (i.e., PETMP) to EGDT (di-thiol monomer) resulted in higher Tg, since having more PETMP leads to a more rigid polymer, and was consistent with dynamic mechanical analysis (DMA) [15] (Supporting Figure S4). Finally, the water contact angle was between 64° to 82°, suggesting a relatively hydrophilic surface.

### 3.2. A systematic Study on Polymer Mass Loss Profile

#### 3.2.1. The Effect of Crosslinker Ratio (Polymer Composition) on Mass Loss Profile

**Figure 1:**
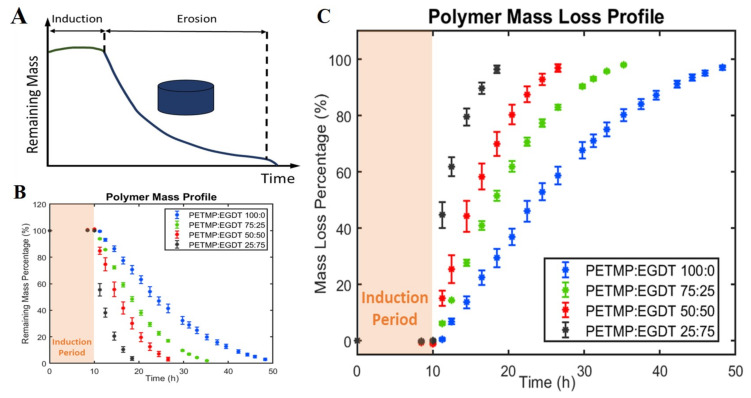
The effect of crosslinking ratio (polymer compositions) on mass loss profiles. (**A**) Schematic mass loss profile of thiol-ene polyanhydrides showing an induction period, and subsequent erosion. (**B**) Remaining mass percentage and (**C**) fractional mass loss percentage of thiol-ene-based polyanhydrides. Increasing the EGDT in polymer networks leads to faster erosion rates. However, changing the mole ratios of PETMP over EGDT did not change the induction period. All experiments were conducted at 25 °C.

Understanding the mass loss profile is needed for determining the erosion rate, which is ideally proportional to the release rate of loaded drugs. The typical mass loss profile of this class of PAHs is shown in Figure 1A. This profile is divided into an induction period and the erosion period [16]. When polymers are immersed in the aqueous media, they absorb some water (induction period) without mass loss and then start eroding until the completion of the polymer mass loss (erosion period). The mass loss data for all four polymers prepared with different crosslinker ratios is collectively shown in Figure 1B,C. After 10 h of induction period [14], the total erosion times ranged from 20 h to 50 h. As expected, a decrease in the PETMP:EGDT crosslinker ratio resulted in a shorter total erosion time without affecting the induction period. The slope of each mass loss curve, which is the erosion rate, is not the same for the different PAHs. The primary reason for differences in erosion rates is the crosslinking density, which decreases with an increasing ratio of EGDT [17]. The relative hydrophobicity of the four different PAH polymers is another potential reason for different erosion rates. A decrease in the water contact angle value from 82 to 64° with decreased PETMP: EGDT mole ratios form PETMP:EDGT = 100:0 to 25:75 is due to the relatively hydrophilic surfaces for polymers with higher EGDT. The lower crosslinking density with relatively more hydrophilic surface leads to faster erosion and, consequently, shorter erosion time for polymers with more EGDT as shown in Figure 1B.

#### 3.2.2. The Effect of Temperature on Mass Loss

**Figure 2:**
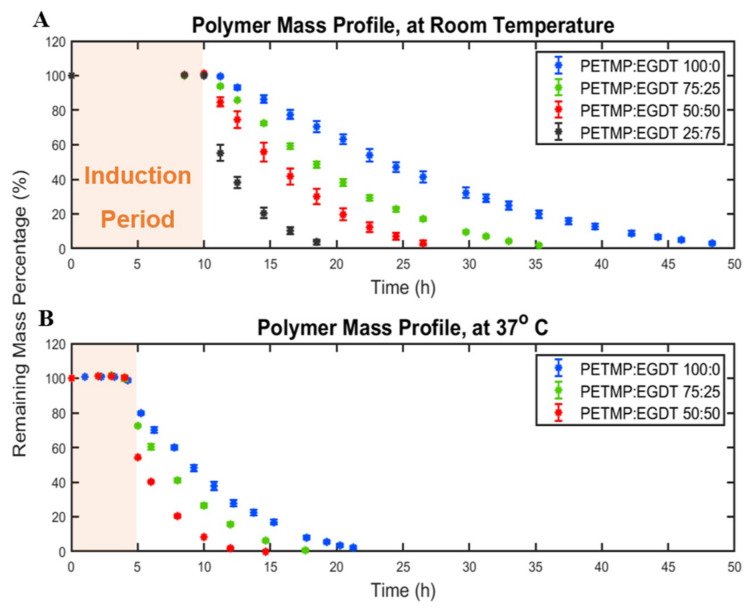
The impact of temperature on polymer mass loss profile. The remaining mass percentage of (**A**) four polymer compositions at room temperature and (**B**) three polymer compositions at 37 °C. Higher temperature leads to shorter induction periods and shorter erosion times.

The reaction that occurs between polymer bonds in contact with the aqueous media drive erosion of biodegradable polymers. Temperature changes the reaction rates and, consequently, the erosion rate of polymers. The effect of temperature on polymer mass loss profiles was investigated by measuring the mass loss of polymers in PBS at ambient temperature (25 °C) and physiological temperature (37 °C), as shown in Figure 2. Two important observations were made: (i) the induction period was reduced from ~10 h to ~5 h at a higher temperature for all polymers, and (ii) the total time needed to erode the PAHs was reduced by 50 % at 37 °C in comparison with 25 °C. In addition, erosion rates were considerably higher at 37 °C than at 25 °C. When the crosslinker mole ratio of PETMP:EGDT was 25:75, the resulting PAH was not stable enough to be evaluated at 37 °C. Although the higher temperature is known to accelerate the degradation rates of biodegradable polymers such as PLA and PLGA [18,19,20], there are no temperature effect studies on this class of photocrosslinkable PAHs. 

#### 3.2.3. The Effect of Shape/Geometry on Mass Loss Behavior

**Figure 3:**
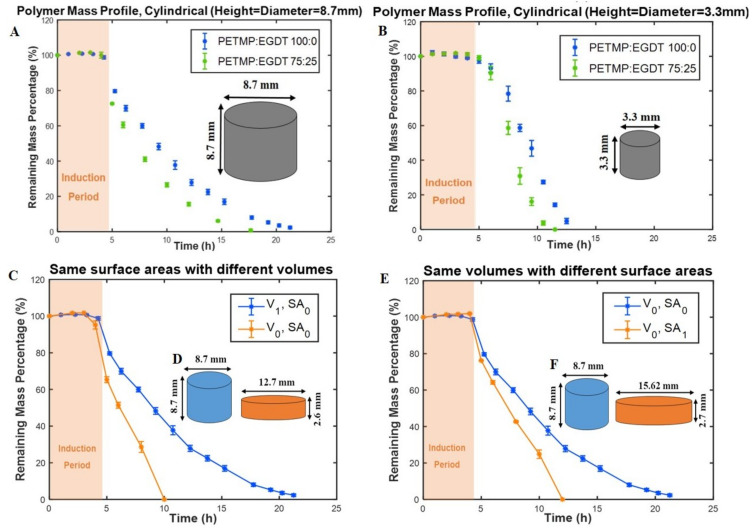
The effect of geometry on the mass loss profile of polymers. Percentage mass remaining for (**A**) big (SA/V ratio = 0.69 mm^–1^) and (**B**) the small (SA/V ratio = 1.82 mm^–1^) cylindrical tablets. (**C**) Remaining mass percentage of two tablets with the same surface areas and different volumes (V_1_ = 1.36 V_0_). (**D**) Schematics of tablet designs and dimensions (SA/V ratio (blue) = 0.69 mm^–1^, and SA/V ratio (orange) = 1.08 mm^–1^). (**E**) Remaining mass percentage of two tablets with the same volumes and different surface areas (SA_1_ = 1.44 SA_0_). (**F**) Schematics of tablet designs and dimensions (SA/V ratio (blue) = 0.69 mm^–1^, and SA/V ratio (orange) = 1.00 mm^–1^). All experiments were conducted at 37 °C.

First, the mass loss data for small cylindrical PAHs (3.3 mm height and 3.3 mm diameter) were compared to the mass loss of the larger size (8.7 mm height and 8.7 mm diameter) tablet having the same composition. This experiment addresses the question of whether the induction period and/or the mass-loss rates are affected by the substantial reduction in the volume of the cylindrical polymers from $\pi \;(1.65^{2}\times 3.3$) to$\;\pi (4.35^{2}\times 8.7)$. Our results show a similar induction period (~5 h) for both small and larger cylindrical samples (Figure 3A,B). The erosion times (from the end of the induction period to the end of the erosion process) for the larger and smaller cylinders decreased from 17 h to 13 h and from 9 h to 6.5 h when PETMP:EGDT = 100:0 was changed to PETMP:EGDT = 75:25, respectively. The reason for shorter erosion times for the smaller cylindrical shape (Figure 3B) is their reduced mass, which was eroded in a shorter time [21]. The similar mass loss profiles observed for both sizes confirm that thiol-ene PAHs maintain their surface erosion behavior even at very small sizes. In PAH biomaterials, there is an understanding that a critical size dimension is needed for surface erosion to delineate from bulk erosion of the same materials [5]. Since the smallest capsule size we fabricated was 3.3 mm × 3.3 mm, our results indicate the high potential of this type of PAHs to be used for various applications that require small size surface-erodible DDSs such as mini-tablets to benefit pediatric patients [22].

The effect of the tablet surface area, volume, and SA/V ratio on the mass loss rates and induction periods of two cylindrical tablets with the same polymer compositions were also studied. The induction periods for the tablets with the same surface areas and different volumes were similar (~5 h), while the total erosion time for the tablet with lower volume was shorter. The reason is that the mass of the smaller volume tablet is less than the larger volume tablet. Although both tablets had similar induction periods, the cylindrical polymer with higher volume has taken longer to erode, with a slower remaining mass loss percentage rate, as shown in Figure 3C,D.

To explore the effect of tablet volumes at a constant surface area on mass loss profiles, two cylindrical tablets were designed and fabricated with the same volumes but different surface areas. The mass loss data for both tablets show similar induction periods; however, the mass-loss rate for the tablet with 44 % higher surface area was faster. Although the total mass of both tablets was initially the same in this case, the tablet with a higher surface area exposed to PBS showed a faster mass loss rate and, consequently, higher erosion rate (Figure 3E,F). This also suggests that the same total amount of drugs can be delivered at different rates by changing the surface areas of the tablet and keeping the volumes constant.

**Figure 4:**
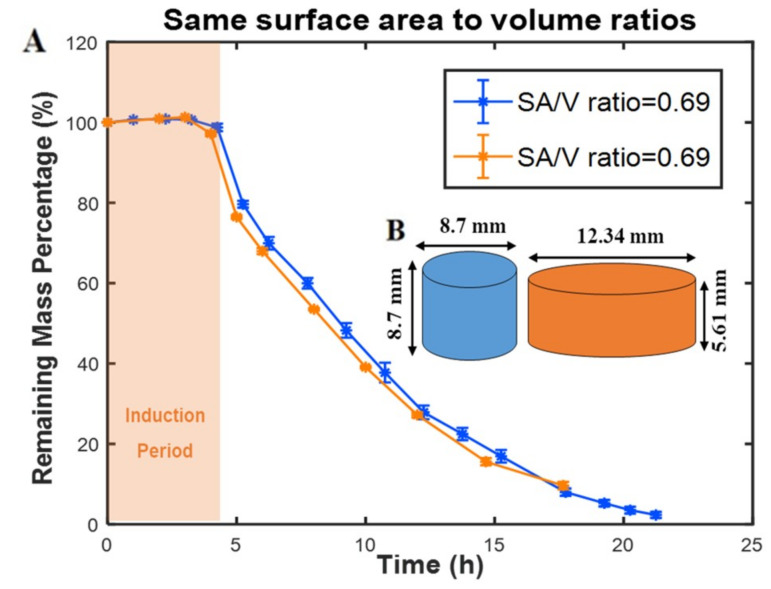
The effect of geometry on the mass loss profile of PAHs. (**A**) Remaining mass percentage of two tablets with the same SA/V ratio while both surface areas and volumes are different. (**B**) Schematics of tablet designs and dimensions. All experiments were conducted at 37 °C.

To study the effect of SA/V ratio, two tablets with different surface areas and volumes were fabricated. For both tablets, the SA/V ratio was kept at 0.69mm^–1^. Although one of the tablets (the orange tablet in Figure 4) had a higher surface area and higher total volume, the mass loss data of both tablets closely follow each other with similar mass loss percentage rates, as shown in Figure 4A. In addition, no induction time changes were observed between the two tablets; however, the mass-loss rate for a polymer with higher surface area and lower volume was faster. These results demonstrate the importance of the SA/V ratio of tablets for determining the mass loss percentage rates. Recently, the importance of the SA/V ratio of tablets is recognized as an essential parameter in drug delivery. For example, Goyans et al. showed that the fractional drug release from an erosion-mediated controlled-release tablet made of polyvinyl alcohol (PVA) filaments was only dependent on the SA/V ratio rather than the surface area or the volume separately [23]. Martinez et al. [24] performed a dissolution test on various tablet geometries with the same SA/V ratios. Tablets with similar SA/V ratios resulted in a similar fractional drug release rate, while tablets with same surface areas showed different fractional release rates. They also showed an increase in dissolution rates of tablets by increasing the SA/V ratio of tablets, which were made of polyethylene glycol diacrylate (PEGDA). Similar results have been obtained for a controlled-release tablet made of hydroxypropylmethylcellulose (HPMC) [25].

#### 3.2.4. The Effect of pH on Erosion Profiles

**Figure 5:**
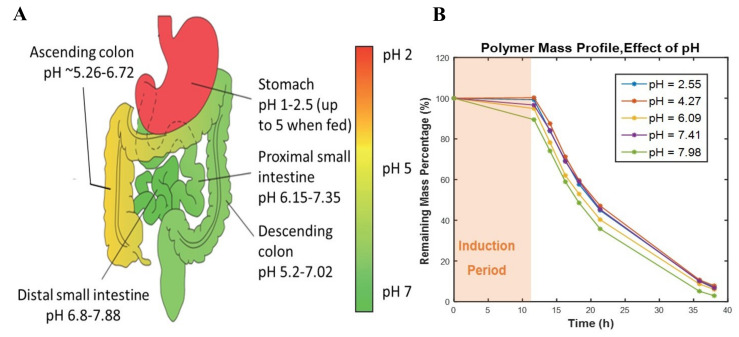
Impact of pHs on mass loss profile of polymers. (**A**) Schematic representation of GI tract pathway with various pHs. Reproduced from Ref [30] with permission. (**B**) Percentage of remaining PAH mass at different pHs.

When biodegradable polymers are used for oral drug delivery, they experience different pHs through the GI tract, and therefore investigating the impact of different pH on erosion behavior of polymers that have potential applications in oral tablets is of high importance. Oral tablets also reside in different parts of the GI tract for specific time intervals at various pHs ranging from 1-2.5 in the stomach to 7.88 at the distal small intestine (Figure 5A). Therefore, the effect of pH on the mass loss profile of a thiol-ene polyanhydride was studied. The induction periods and mass loss rates were similar for all samples except for the one at pH=7.89, which showed a shorter induction period and a faster mass loss rate (Figure 5B). PAHs undergo more rapid erosion by both base- and water-catalyzed hydrolysis than in acid-catalyzed conditions. The erosion involves the addition of a base to the carbonyl carbon leading to the generation of the anhydride through the removal of hydroxide anion—a process that is faster in alkaline conditions. The faster mass loss rate of the polymer in alkaline solution is in close agreement with studies that showed an acceleration in the degradation of biodegradable polymers in a more alkaline environment [8,26,27,28,29]. Considering the induction period, which lasted a few hours, and based on the mass loss data obtained from different pHs, it can be inferred that a tablet fabricated from this polymer will undergo similar erosion profiles through the GI tract with variable pHs.

#### 3.2.5. The Effect of Mass Transfer and Model Compound on Erosion Behavior

**Figure 6:**
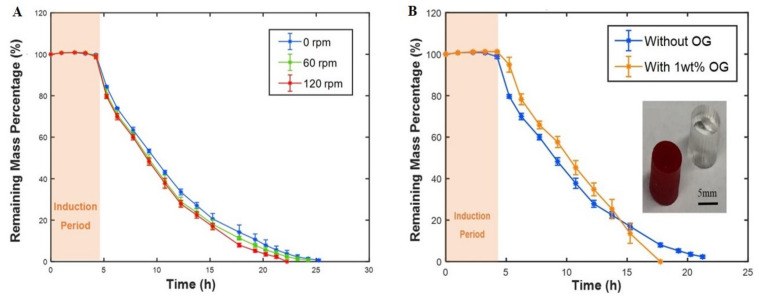
(**A**) Mass transfer effect on the polymer mass loss profile. Different shaking rates (0, 60, 120 rpm) were used. (**B**) Effect of adding the model compound to the polymer on mass loss profile. All experiments were conducted at 37 °C.

To explore the effect of mass transfer on the polymer mass loss profile, various shaking rates were used. Different shaking rates create different convective forces that can change the erosion rates by removing the degradation products from the tablets. The induction time was similar for the different shaking rates. However, there is a trend towards an accelerated erosion rate with increasing the shaking (Figure 6A). Our findings are consistent with the reports of Shieh et al. [31], who found a similar trend for a different type of PAH, namely, poly (Fatty Acid Dimer: Sebacic Acid) poly(FAD:SA). The effect of adding drugs to the polymer may change some physicochemical properties of polymers causing changes in polymer degradation behavior. To elucidate this, we compared pure and dye-loaded tablets (Figure 6B). As can be seen, there was no considerable difference in the induction time, but the mass-loss rate was higher for model compound-loaded PAH tablets. More specifically, during the final five hours of the erosion period, the polymer containing the model compound eroded rapidly (see the orange line in Figure 6B). This is potentially due to the lower crosslink density in the center of the dye-loaded polymer suggesting a reduction in the penetration depth of the UV-light during crosslinking. To obtain the same behavior for the dye-loaded polymer as the pure polymer, longer crosslinking times or higher intensity UV sources may be needed.

#### 3.2.6. Pre-Erosion of PAHs to Eliminate the Induction Time

**Figure 7:**
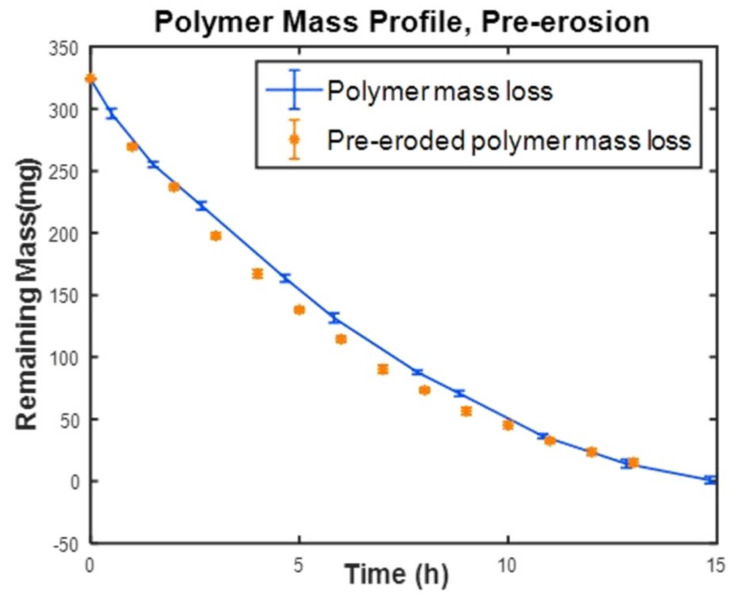
Pre-erosion of PAH tablets to eliminate the induction period. The tablets were pre-eroded for 8h and vacuum-dried before subjected to mass loss experiments at 37 °C.

The induction period (or lag time) during which the PAH polymer only absorbs some water lowers the predictability of the polymer mass loss and therefore is undesired. In addition, for treating some diseases, the release of the drug must start as soon as the patient swallows the tablet. Therefore, waiting for 5 h induction after taking the tablet may not be practical. We suspected that the induction period could be eliminated by pre-erosion without affecting the release profile. To test this speculation, cylindrical tablet samples prepared from PNA:PETMP:EGDT = 100:75:25 were immersed in PBS for 8 h at 37 °C. The samples were removed from the solution after the induction period and when the erosion had just been started. After drying under vacuum for 10 h, the samples were placed again in PBS, and the mass loss measurements were carried out. When the mass loss profile of the pre-eroded samples was compared with samples that were not pre-eroded, it showed a similar trend without any appreciable difference (Figure 7). Thus, when the polymer is placed in PBS after pre-erosion and vacuuming, the polymer continues the erosion from where it left off, instead of undergoing a second stage induction period. This finding shows that the induction period can be eliminated for drug-loaded photocrosslinked PAHs by means of pre-erosion. Elimination of the lag phase (induction time) by pre-erosion is thought to be a desirable feature of DDS, as demonstrated in another study for a different type of PAH [7]. However, the benefit of the current study to this cited study [7] was our ability to carry out the pre-erosion for a shorter time at physiologic temperature (37 °C) instead of the reported 60 °C.

#### 3.2.7. Non-Linear Fitting of Mass Loss Data

**Figure 8:**
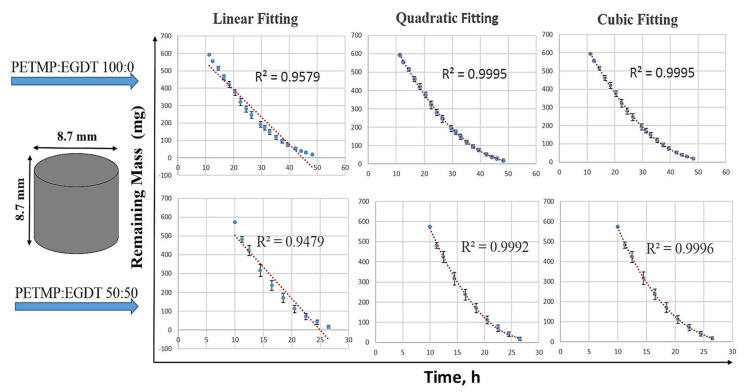
Fitting mass loss data for two cylindrical polymers with initial mole ratios of PNA:PETMP: EGDT = 100:100:0 and 100:50:50 as a function of time. The linear, quadratic, and cubic fits and their R^2^ show that the linear equation is not the best-fitted equation and the mass loss is not changing linearly.

The mass loss data collected for cuboid and cylindrical polymers were used for fitting with the linear, quadratic, and cubic polynomials. The collected data during the erosion period were used for curve fitting since there are no considerable changes during the induction period. Shown in Figure 8 is an example of a cylindrical shape erosion data in a non-linear quadratic best fit. Curve fitting for other cuboid and cylindrical polymers of different composition are shown in Supporting Figures S5 and S6. Table 1 summarizes the mathematical equations and the R^2^ values of each fit to the mass loss data for a cuboid and cylindrical PAH tablets. 

### 3.3. Correlation of Mass Loss Data to Release Kinetic Models 

Mathematical modeling is a useful tool to accelerate the development of controlled release of drugs or biomolecules and play an important role in understanding the physicochemical mechanisms. As drug release rate from surface eroding PAHs follows the polymer mass loss profile (i.e., it is proportional to the erosion rate of the polymer), release kinetic models fitting on the mass loss data for thiol-ene PAHs were examined. There are several release kinetic models available, among which zero-order, first-order, Higuchi, Korsmeyer-Peppas, Hixson-Crowell, Hopfenberg, and Weibull are the most commonly used for describing the release profile from polymeric systems [32,33]. 

The zero-order kinetic model describes a diffusion-based release system where the drug is released at a constant rate regardless of its concentration. Equation (3) shows the zero-order release kinetic model where $C_{0}$ is the initial amount of the drug in the solution, $C_{t}$ is the total released drug until time t, and $K_{0}$ is the zero-order rate constant. Most of the transdermal systems and some tablet matrix systems containing drugs with low solubility are examples of zero-order release kinetics applications [34]. Here, the zero-order model described in Equation (3) fitted to the measured cumulative mass eroded over time is used.

(3) $$C_{t}=C_{0}-K_{0}t$$

The first-order model describes the concentration-dependent diffusion-based release behavior. Equation (4) shows the first-order kinetics model where $C_{t}$ is the total released drug until time t, and K is the first-order rate constant. Equation (4) can be reorganized to another form of first-order release kinetics model which is shown in Equation (5), where $C_{0}$ is the initial concentration of drug in the solution. The main application of this model is for water-soluble drug-loaded dosage forms in porous structures [35]. The log of the remaining mass percentage versus time to the first-order model (Equation (4)) gives a straight line with a slope of $\frac{-Kt}{2.303}$.

(4) $$\frac{dC_{t}}{dt}=-KC$$

(5) $$logC_{t}=logC_{0}-\frac{Kt}{2.303}$$

The first and most commonly used mathematical model for describing the drug release rate from matrices was developed by Higuchi [36]. Initially, the model was only developed for planar systems, which was later modified to a more complicated equation that considers porous polymers in different geometries [37]. The basic Higuchi model is shown in Equation (6), where C is the amount of the drug release per unit area of the matrix, D is the diffusion coefficient for the drug in the matrix, qt is the total amount of drug in a unit volume of matrix, $C_{S}$ is the dimensional solubility of drug in the polymer matrix, and t is the time. Higuchi model can be simplified to Equation (7), which relates the cumulative drug release (the total released drug until time t) to the square root of time by Higuchi constant ($K_{H}$). The percentage of the fractional cumulative mass eroded versus square root of time was fitted to Equation (7).

(6) $$C={\left[D\left(2qt-C_{S}\right)C_{S}t\right]}^{\frac{1}{2}}$$

(7) $$\frac{C_{t}}{C_{\infty }}=K_{H}t^{\frac{1}{2}}$$

The power law is a semi-empirical model that relates drug release with time exponentially, as described by Equation (8). The Korsmeyer-Peppas equation [38] was developed for drug release from hydrophilic polymers as shown in Equation (9), where $\frac{C_{t}}{C_{\infty }}$ is the drug release fraction at time = t, K is the rate constant, and n is the release exponent. By deriving the release exponent n (ranging from 0 to 1), the release mechanism of the polymeric system with certain geometry can be interpreted [39]. To find the release mechanism, the first 60% of mass loss data were used. Here, the log of the fractional mass eroded over log of time were fitted to Equation (9).

(8) $$\frac{C_{t}}{C_{\infty }}=Kt^{n}$$

The above equation can also be written as:

(9) $$log\frac{C_{t}}{C_{\infty }}=logK+n\;log\;t$$

The Hixson-Crowell model, Equation (10), was developed with the argument that the surface area of a group of particles is proportional to the cubic root of their volume [40].

(10) $$C_{0}{}^{\frac{1}{3}}-C_{t}{}^{\frac{1}{3}}=K_{HC}t$$

where $C_{0}$ is the initial amount of the drug, $C_{t}$ is the total amount of the drug released by time t, and $K_{HC}$ is the Hixson-Crowell rate constant. This model describes the systems in which the surface area and diameter of particles or tablets change over time. In this study, the cubic root of the fractional mass eroded versus time was fitted to the Hixson-Crowell model.

The model developed by Hopfenberg describes drug release from erodible systems with different geometries. In the Hopfenberg model, the release rate is proportional to the surface area of the system. Equation (11) shows the Hopfenberg empirical equation where $C_{\infty }$ is the initial amount of drug-loaded in the system, $C_{t}$ is the total amount of drug released by time t, $K_{0}$ is the rate constant of surface erosion process, a is the half-thickness (half-thickness in case of slab or radius in case of cylinders or spheres), $C_{l}$ is the initial loaded drug in the system, and n is the release exponent (n = 1 for slab, n = 2 for cylindrical, and n = 3 for spherical geometries) [41]. In this study, the fractional mass eroded (mass loss) versus time was fitted to the Hopfenberg model with n = 1 for cubic and n = 2 for cylindrical tablets.

(11) $$\frac{C_{t}}{C_{\infty }}=1-{\left[1-\frac{K_{0}t}{C_{l}a}\right]}^{n}$$

The Weibull kinetic model is another empirical model that can be used for dissolution and releasing drugs from oral dosage forms [42]. The Weibull model is shown in Equation (12) where $C_{0}$ is the total amount of drug loaded, C is the total amount of the drug released by time t, T is the lag time, a is the scale parameter, and b is the release curve shape. The fractional mass eroded (mass loss) versus time were fitted to Weibull model (Equation (12)).

(12) $$C=C_{0}\left[1-e^{\frac{-{\left(t-T\right)}^{b}}{a}}\right]$$

**Figure 9:**
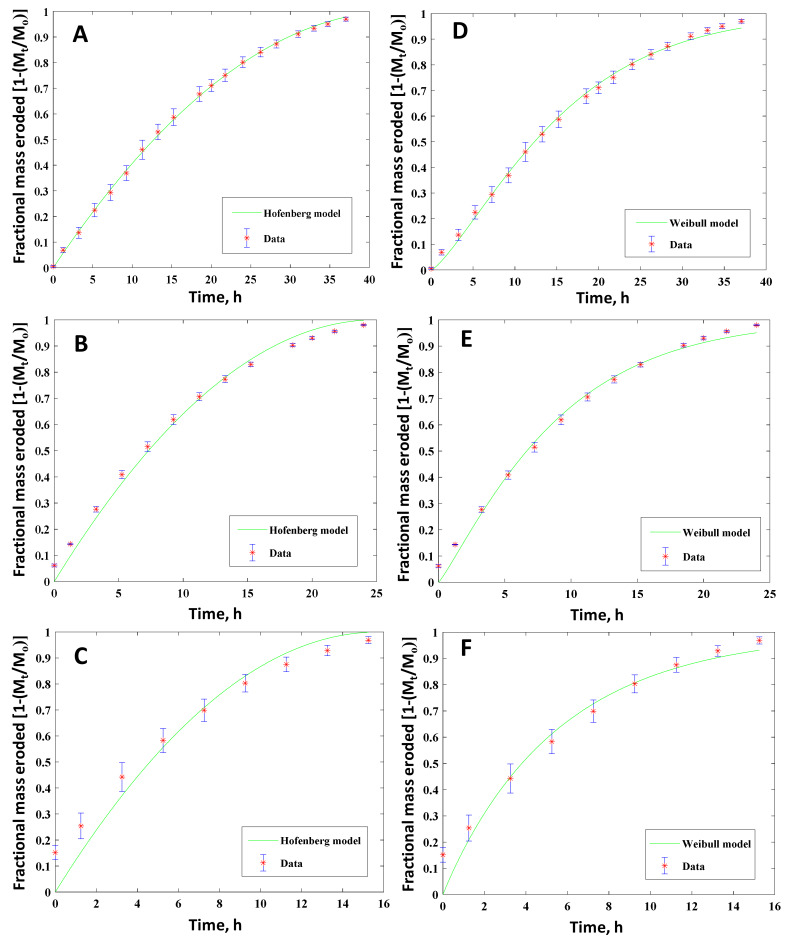
Fractional mass eroded as a function of time for cylindrical PAH tablets with different crosslinking ratios in PNA:PETMP:EGDT systems. Mole ratios of 100:100:0 (**A,D**), 100:75:25 (**B,E**) and 100:50:50 (**C,F**). Best fitted kinetic models with red dots (experimental data) and green lines are the fitted curves for Hopfenberg (**A–C**) and Weibull (**D–F**) release kinetic models.

**Figure 10:**
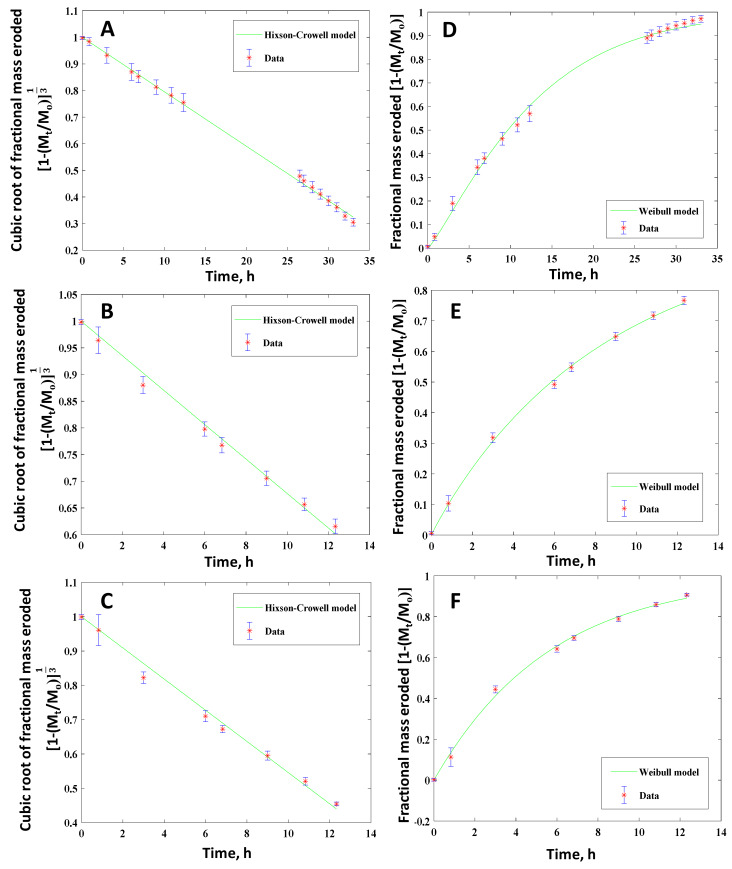
Fractional mass eroded as a function of time for cuboid PAH tablets with different crosslinking ratios in PNA:PETMP:EGDT systems. Mole ratios of 100:100:0 (**A**,**D**), 100:75:25 (**B**,**E**) and 100:50:50 (**C**,**F**). Best fitted kinetic models with red dots (experimental data) and green lines are the fitted curves for Hixson-Crowell (**A**–**C**) and Weibull (**D**–**F**) release kinetic models. Hixson-Crowell kinetic model, as one of the best-fitted models, is developed for erosion mechanism.

In the current study, the experimental mass loss data were fitted to the above seven mathematical models using MATLAB MathWorks software. The coefficient of correlation R^2^ and the root mean squared error (RMSE) was used for verifying the fitting accuracy in order to find the best-fitted model. A model with the highest R^2^ (correlation coefficient closest to 1) and the minimum RMSE (equal to highest concentrated data around the line of fit) was considered as the best-fitted model. The regression coefficient, R^2^, and the best-fit model parameters are reported. Figure 9 shows the selected best fitted kinetic models for the cylindrical polymers with two different compositions (PETMP:EGDT = 100:0, 75:25). As summarized in Table 2, the Hopfenberg and Hixson-Crowell models gave the best results for cylindrical samples. However, the Hixson-Crowell and Weibull models yielded the best fit for cuboid-shaped samples for the same PAHs (Figure 10 and Table 3), suggesting the importance of shape factors in mass loss kinetics. Since the Hixson-Crowell and Hopfenberg models are developed for erosion mechanisms rather than diffusion-controlled systems, the experimental erosion data is consistent with the models. The results of curve fitting to other models can be found in Supporting Figures S7–S11 for both cylindrical and cuboid polymers. Using the derived parameters form model fitting, the release of the drug through these polymers can be predicted. 

## 4. Discussion

In this study, we showed the effect of several factors on the mass loss behavior of photocrosslinked PAHs. The effect of polymer composition on mass loss profiles (both the induction and the erosion period) was studied by changing the initial mole ratios of the crosslinkers (PETMP to EGDT). Although changing the polymer compositions did not affect the induction period, an increased initial mole ratio of EGDT to PETMP resulted in a shorter total erosion time. The effect of the tablet geometry was examined by first comparing the mass loss profiles of small cylindrical tablets with a larger one made of the same polymers. Similar induction periods and erosion patterns were observed for the two tablet geometries, indicating that the thiol-ene PAHs maintain their surface erosion behavior even at very small dimensions (e.g., 3 mm), similar to other types of PAHs [5]. This shows the potential of this type of PAHs to be used in small-size controlled DDSs. In another set of experiments, the impact of the surface area, volume, and SA/V ratio on the erosion behavior of the tablets were examined. While the induction period was not affected by these parameters, the mass loss for a polymer with a higher surface area and/or a lower volume was shown to be faster. Polymers with the same SA/V ratios showed similar mass loss percentage rates despite their dissimilar volumes and surface areas. The importance of SA/V ratio in the degradation behavior of tablets made of other biodegradable polymers has been shown before [24,25].

The impact of parameters such as the temperature, pH, and mass transfer on the mass loss profile of thiol-ene PAHs was also studied. A decrease in the induction period (from 10 to 5 h) was observed in the mass loss profile of the polymers when the temperature increased (from 25°C to 37 °C). The erosion was considerably faster at higher temperatures. The findings are in agreement with other studies showing that the degradation of biodegradable polymers is accelerated at elevated temperatures [18,19,20]. The induction times and mass loss rates were similar for PAHs at different pH, except for the one in pH = 7.89, which showed a shorter induction period and faster mass loss rate. The increased mass loss rate of the polymer in the alkaline solution is in agreement with results from other studies that showed an acceleration in the degradation of biodegradable polymers in alkaline environments [27,28,29]. The induction period and mass loss rate of polymers were affected by shaking rates (0, 60, 120 rpm), with a lower erosion rate observed at static conditions. These findings indicated the effect of the shaking rate on the mass loss profile of this type of PAHs which is similar to the behavior observed for another type of polyanhydride with the slightly slower erosion rate in the solution with a lower shaking rate [31]. Adding hydrophilic compounds (such as the neutral form of the lidocaine) to the polymers might decrease the crosslinking density, which leads to a faster mass loss. Although the dispersion of various amount of lidocaine (1, 2, and 3 wt. %) in other cross-linked thiol-ene PAHs caused slightly faster mass loss [17], dispersion of the 1 wt. % model compound Orange G in the present PAHs did not make a considerable difference in the mass loss rates of the tablets.

The feasibility of eliminating the undesired induction period (lag time) of thiol-ene PAHs was studied by pre-eroding the polymers in PBS [7]. The successful elimination of the pre-erosion can be attributed to a decrease in the hydrophobicity of the polymer surface caused by the hydrolysis that occurs when the tablet is in contact with PBS in the pre-erosion period. Although the temperature was the only parameter that could affect the induction period substantially, the mass-loss rate was influenced by most of the parameters studied, including the polymer composition and geometry as well as the temperature, pH, and shaking rate of the PBS solution during the experiments.

This study also documented data on the mass loss behavior of the cylindrical and cuboid thiol-ene PAHs by fitting their experimental mass loss data to linear, quadratic, and cubic functions. Even though a linear mass loss profile is commonly reported for surface eroding polymers [3,5], the mass loss data of the present polymers more closely followed at least a quadratic function. The geometry of the tablets is attributed to be the reason behind this non-linear behavior of the mass loss data. Polymers in the form of a slab showed a linear mass loss while cubes or cylinders made of the same polymer show more complicated patterns [16]. The release kinetic models fitting was also conducted to further investigate the mass loss profiles of the polymers. With the highest R^2^ and the lowest RMSE, the Hixson-Crowell, Hopfenberg, and Weibull models best fitted the cylindrical and cuboid mass loss data. These best-fitted models describe the erosion mechanism of the systems rather than the diffusion mechanisms that govern the other kinetic models used for data fitting.

To further advance this study, the mass loss measurements can be conducted in PBS solutions with changing pHs over time [43] to mimic the conditions that a solid oral dosage form would experience through the GI tract. In this regard, additional mass loss experiments conducted in more physiologically relevant environments could help provide more accurate predictions of the in vitro and, ultimately, in vivo erosion behavior of these polymers.

## Figures and Tables

**Table 1:** Mass loss data fitting. linear, quadratic, and cubic polynomials were fitted to the mass loss data of four polymers with different compositions of monomers in two different shapes.

Geometry	Mole Ratio PNA:PETMP:EGDT	Fitting Curve	(R^2^)
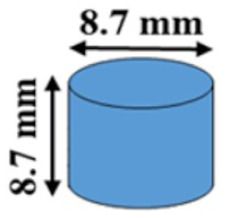	100:100:0	Linear	0.9579
		Quadratic	0.9995
		Cubic	0.9995
	100:75:25	Linear	0.9448
		Quadratic	0.9995
		Cubic	0.9995
	100:50:50	Linear	0.9479
		Quadratic	0.9992
		Cubic	0.9996
	100:25:75	Linear	0.8318
		Quadratic	0.9673
		Cubic	0.9940
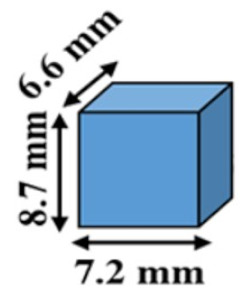	100:100:0	Linear	0.9647
		Quadratic	0.9983
		Cubic	0.9988
	100:75:25	Linear	0.9656
		Quadratic	0.9978
		Cubic	0.9993
	100:50:50	Linear	0.929
		Quadratic	0.993
		Cubic	0.9982
	100:25:75	Linear	0.8569
		Quadratic	0.8689
		Cubic	0.9998

**Table 2:** Release kinetic models fitting for a cylindrical tablet. The models with their equations, mole ratios of monomers, R^2^, and derived parameters are shown in this table.

Model	Mathematical Equation	PNA:PETMP: EGDT	(R^2^)	Estimated Parameters
**Zero-order**	$C=C_{0}-K_{0}t$	100:100:0	0.9217	k_0_ = −18.34 (−19.55, −17.14)
		100:75:25	0.8953	k_0_ = -26.5 (−29.1, −23.89)
		100:50:50	0.9226	k_0_ = -35.23 (−39.29, −31.18)
		100:25:75	0.9001	k_0_ = -45.09 (−55.85, −34.33)
**First-order**	$logC=logC_{0}-\frac{Kt}{2.303}$	100:100:0	0.9370	k = 0.07648 (0.07046, 0.0824)
		100:75:25	0.9451	k = 0.1346 (0.1222, 0.1471)
		100:50:50	0.9624	k = 0.1992 (0.1804, 0.218)
		100:25:75	0.8867	k = 0.458 (0.3527, 0.5633)
**Higuchi**	$\frac{C_{t}}{C_{\infty }}=K_{H}t^{\frac{1}{2}}$ ($n=\frac{1}{2}$)	100:100:0	0.9494	K_H_ = 15.54 (14.72, 16.36)
		100:75:25	0.9770	K_H_ = 20.36 (19.51, 21.22)
		100:50:50	0.9628	K_H_ = 25.52 (23.89, 27.15)
		100:25:75	0.2902	K_H_ = 39.84 (30.07, 49.6)
**Korsmeyer-Peppas (Power Law)**	$log\frac{C_{t}}{C_{\infty }}=logK+n\;logt$	100:100:0	0.8318	logk = 0.2571 (−0.1998, 0.714) n = 1.367 (0.8176, 1.916)
		100:75:25	0.9514	logk = 0.938 (0.7709, 1.105) n = 0.9001 (0.6661, 1.134)
		100:50:50	0.9555	logk = 1.259 (1.104, 1.414) n = 0.7061 (0.4262, 0.986)
		100:25:75	0.8341	logk = 1.695 (0.976, 2.414) n = 0.4218 (−1.968, 2.812)
**Hixson-Crowell**	$C_{0}{}^{\frac{1}{3}}-C_{t}{}^{\frac{1}{3}}=K_{HC}t$	100:100:0	0.9954	k= 0.01771 (0.01738, 0.0180)
		100:75:25	0.9983	k = 0.02973 (0.02931, 0.0301)
		100:50:50	0.9856	k = 0.04508 (0.0428, 0.04736)
		100:25:75	0.5750	k = 0.1009 (0.06404, 0.1377)
**Hopfenberg**	$\frac{C_{t}}{C_{\infty }}=1-{\left[1-\frac{K_{0}t}{C_{l}a}\right]}^{n}$ (n=2)	100:100:0	0.9994	k = 3.146 (3.113, 3.179)
		100:75:25	0.9901	k = 5.366 (5.073, 5.659)
		100:50:50	0.9364	k = 8.295 (6.895, 9.695)
		100:25:75	0.9508	k = 18.21 (14, 22.42)
**Weibull**	$C=C_{0}\left[1-e^{\frac{-{\left(t-T\right)}^{b}}{a}}\right]$	100:100:0	0.9976	b = 1.294 (1.233, 1.354) a = 37.26 (30.76, 43.76)
		100:75:25	0.9926	b = 1.146 (1.023, 1.268) a = 12.66 (8.982, 16.33)
		100:50:50	0.9591	b = 0.974 (0.6625, 1.286) a = 5.301 (2.068, 8.534)
		100:25:75	0.1248	b = 0.6196 (−1.627, 2.866) a = 1.221 (−1.886, 4.328)

**Table 3:** Release kinetic models fitting for cuboid polymers. The models with their equations, mole ratios of monomers, R^2^, and derived parameters are shown in this table.

Model	Mathematical Equation	PNA:PETMP: EGDT	(R^2^)	Estimated Parameters
**Zero-order**	$C=C_{0}-K_{0}t$	100:100:0	0.9114	k_0_ = -15.53 (−16.76, −14.3)
		100:75:25	0.9412	k_0_ = -32.12 (−35.7, −28.53)
		100:50:50	0.8874	k_0_ = -38.7 (−44.57, −32.84)
		100:25:75	0.6547	k_0_ = -124.9 (−265.9, 16.07)
**First-order**	$logC=logC_{0}-\frac{Kt}{2.303}$	100:100:0	0.9690	k = 0.09395 (0.08832, 0.09958)
		100:75:25	0.9990	k = 0.1163 (0.1144, 0.1181)
		100:50:50	0.9936	k = 0.1818 (0.1742, 0.1894)
		100:25:75	0.9085	k = 0.6756 (0.2746, 1.077)
**Higuchi**	$\frac{C_{t}}{C_{\infty }}=K_{H}t^{\frac{1}{2}}$ ($n=\frac{1}{2}$)	100:100:0	0.9803	K_H_ = 16.81 (16.19, 17.43)
		100:75:25	0.9782	K_H_ = 21.06 (19.65, 22.47)
		100:50:50	0.9805	K_H_ = 25.89 (24.28, 27.5)
		100:25:75	0.8912	K_H_ = 54.5 (25.18, 83.81)
**Korsmeyer-Peppas (Power Law)**	$log\frac{C_{t}}{C_{\infty }}=logK+n\;logt$	100:100:0	0.8680	logk = 0.2705 (−0.1763, 0.7174 n = 1.477 (0.9015, 2.052)
		100:75:25	0.7553	logk = 0.3148 (−0.7788, 1.408) n = 1.89 (-0.08656, 3.867)
		100:50:50	0.7067	logk = 0.04847 (−2.358, 2.455) n = 2.762 (−2.652, 8.177)
		100:25:75	0.8316	logk = 0.1069 (−5.915, 6.129) n = 3.501 (−16.52, 23.52)
**Hixson-Crowell**	$C_{0}{}^{\frac{1}{3}}-C_{t}{}^{\frac{1}{3}}=K_{HC}t$	100:100:0	0.9984	k = 0.02047 (0.02022, 0.02073)
		100:75:25	0.9923	k = 0.0323 (0.03092, 0.03368)
		100:50:50	0.9902	k = 0.04542 (0.04321, 0.04762)
		100:25:75	0.7724	k = 0.1758 (0.02395, 0.3277)
**Hopfenberg**	$\frac{C_{t}}{C_{\infty }}=1-{\left[1-\frac{K_{0}t}{C_{l}a}\right]}^{n}$ (n=1)	100:100:0	0.9114	k = 3.571 (3.288, 3.854)
		100:75:25	0.9371	k = 7.452 (6.6, 8.304)
		100:50:50	0.8858	k = 8.922 (7.564, 10.28)
		100:25:75	0.5071	k = 31.88 (−6.816, 70.59)
**Weibull**	$C=C_{0}\left[1-e^{\frac{-{\left(t-T\right)}^{b}}{a}}\right]$	100:100:0	0.9973	b = 1.188 (1.119, 1.257) a = 21.34 (17.41, 25.27)
		100:75:25	0.9990	b = 0.9593 (0.8992, 1.019) a = 7.856 (6.872, 8.84)
		100:50:50	0.9973	b = 1.009 (0.8991, 1.12) a = 5.712 (4.485, 6.939)
		100:25:75	0.9796	b = 0.5083 (−3.196, 4.213) a = 0.8737 (−1.289, 3.037)

## Supplementary Materials

The following are available online at https://www.mdpi.com/2073-4360/12/5/1105/s1, **Figure S1**: ATR-FTIR spectra of photocrosslinked polyanhydrides with different initial mole ratios of monomers. **Figure S2:** PXRD patterns of thiol-ene polyanhydrides with different initial mole ratios of crosslinkers. **Figure S3:** TGA traces for four polyanhydrides to check their decomposition temperatures before doing the DSC experiments. **Figure S4:** DSC of four different polymers with initial mole ratios of PNA:PETMP:EGDT equal to: A) 100:100:0. B) 100:75:25. C) 100:50:50. D) 100:25:75. **Figure S5:** Fitting mass loss data for cylindrical tablets. Linear, quadratic, and cubic fitting curves to the mass loss data of PAHs with initial mole ratios of PETMP:EGDT=75:25 and 25:75. The R^2^ values are shown on the graphs. **Figure S6:** Fitting mass loss data for cuboid polymers. Linear, and cubic fitting curves to the mass loss data of PAHs with initial mole ratios of PETMP:EGDT=100:0, 75:25, and 50:50. The R^2^ values are shown on the graphs. **Figure S7:** Cumulative mass eroded as a function of time fitted to zero-order kinetic model for cylindrical and cuboid PAH tablets. **Figure S8:** Log of the remaining mass as a function of time fitted to first-order kinetic model for cylindrical and cuboid PAH tablets. **Figure S9:** Fractional mass eroded percentage as a function of square root of time fitted to Higuchi kinetic model for cylindrical and cuboid PAH tablets. **Figure S10:** Log of the fractional mass eroded percentage as a function of the log(time) fitted to Korsmeyer-Peppas kinetic model for cylindrical and cuboid PAH tablets. **Figure S11:** Cubic root of fractional mass eroded as a function of time for cylindrical (A-C) and cuboids (D-F) PAH tablets with different crosslinking ratios in PNA: PETMP:EGDT systems. Fitted kinetic models with red dots (experimental data) and green lines are the fitted curves for Hixson-Crowell (A-C) and Hopfenberg (D-F) release kinetic models.
