# Supplementary material for: Systematic Studies on Surface Erosion of Photocrosslinked Polyanhydride Tablets and Data Correlation with Release Kinetic Models
Source: Polymers (Basel). 2020 May 12;12(5):1105. doi: 10.3390/polym12051105 (PMC7285269; doi:10.3390/polym12051105)
Supplement: Supplementary file 1 [file polymers-12-01105-s001.pdf]

Supporting Information

Systematic Studies on Surface Erosion of Photocrosslinked Polyanhydride Tablets and Data Correlation with Release Kinetic Models

Armin Geraili [1], Kibret Mequanint [1,2*]

[1] School of Biomedical Engineering, The University of Western Ontario, London Ontario N6A 5B9, Canada
[2] Department of Chemical and Biochemical Engineering, The University of Western Ontario, London Ontario N6A 5B9, Canada

*Corresponding author. E-mail: kmequani@uwo.ca

A

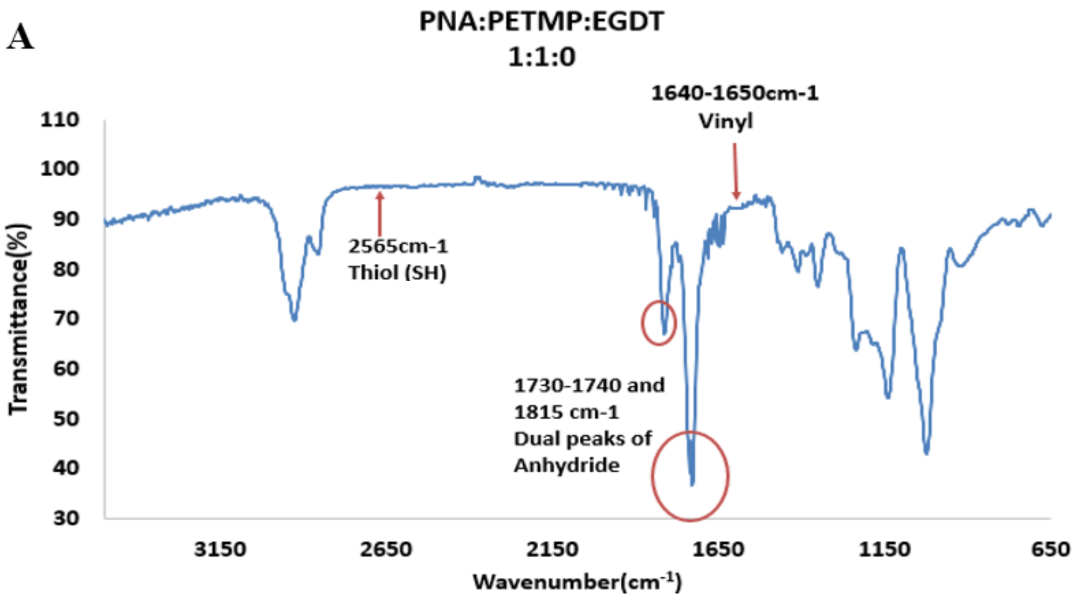

B

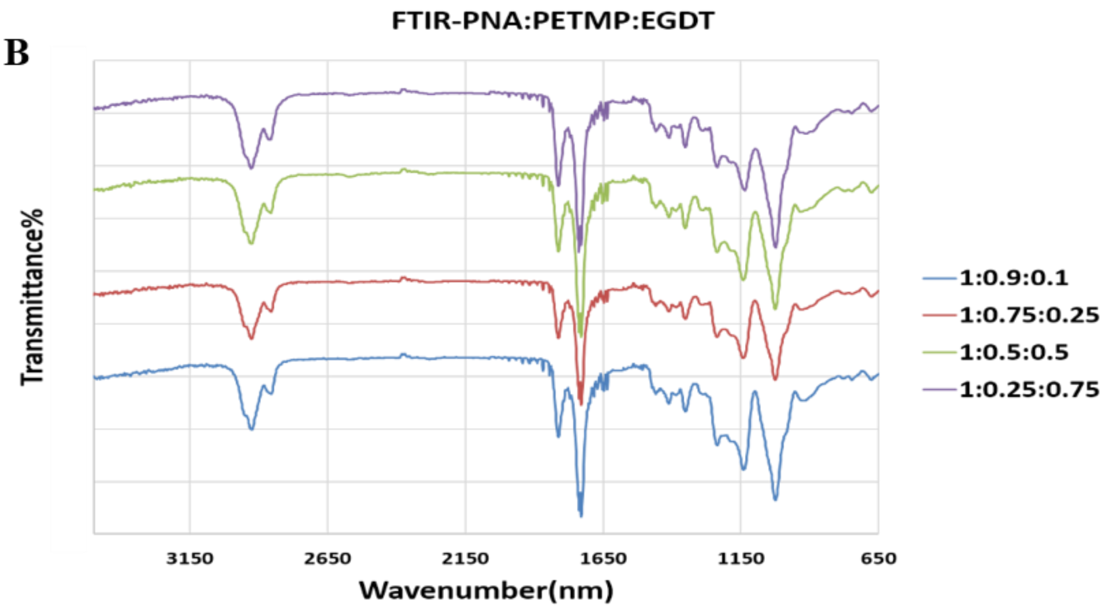

Figure S1: ATR-FTIR spectra of A) PNA: PETMP = 1:1:0 shows the dual peaks of anhydrides and the absence of thiol and vinyl functional groups' peaks. B) The same peaks for other synthesized polyanhydrides with different initial mole ratios of monomers.

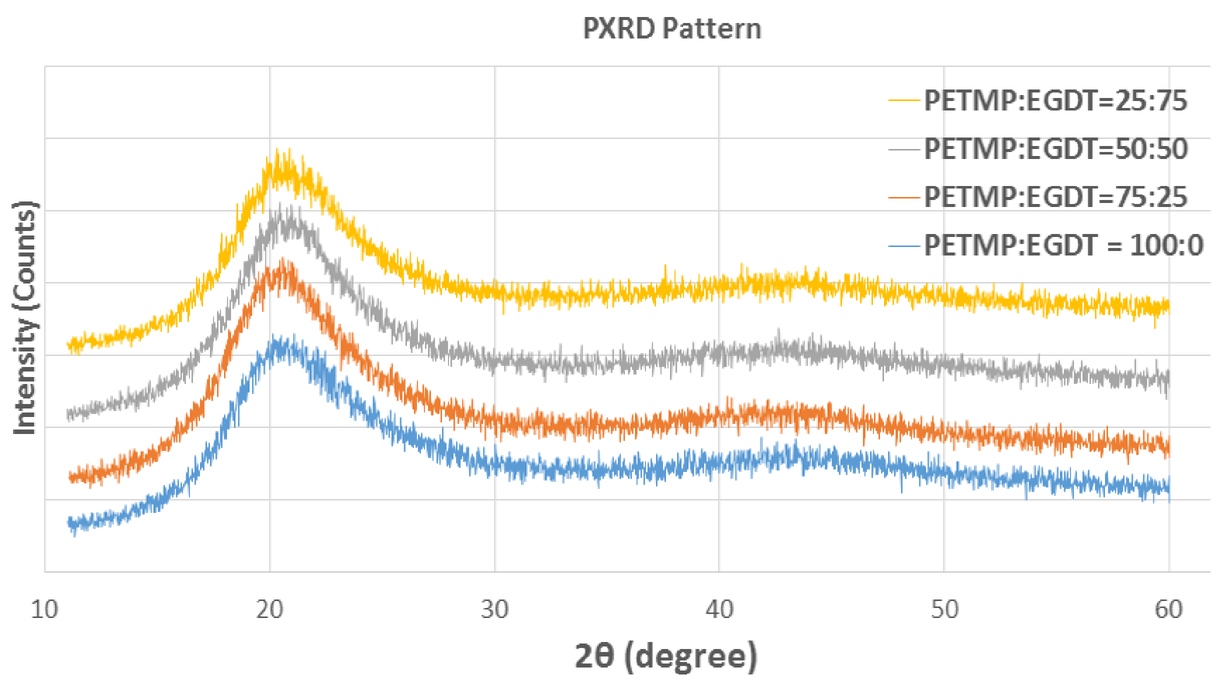

Figure S2: PXRD patterns of thiol-ene polyanhydrides with different initial mole ratios of crosslinkers. traces for four polyanhydrides.

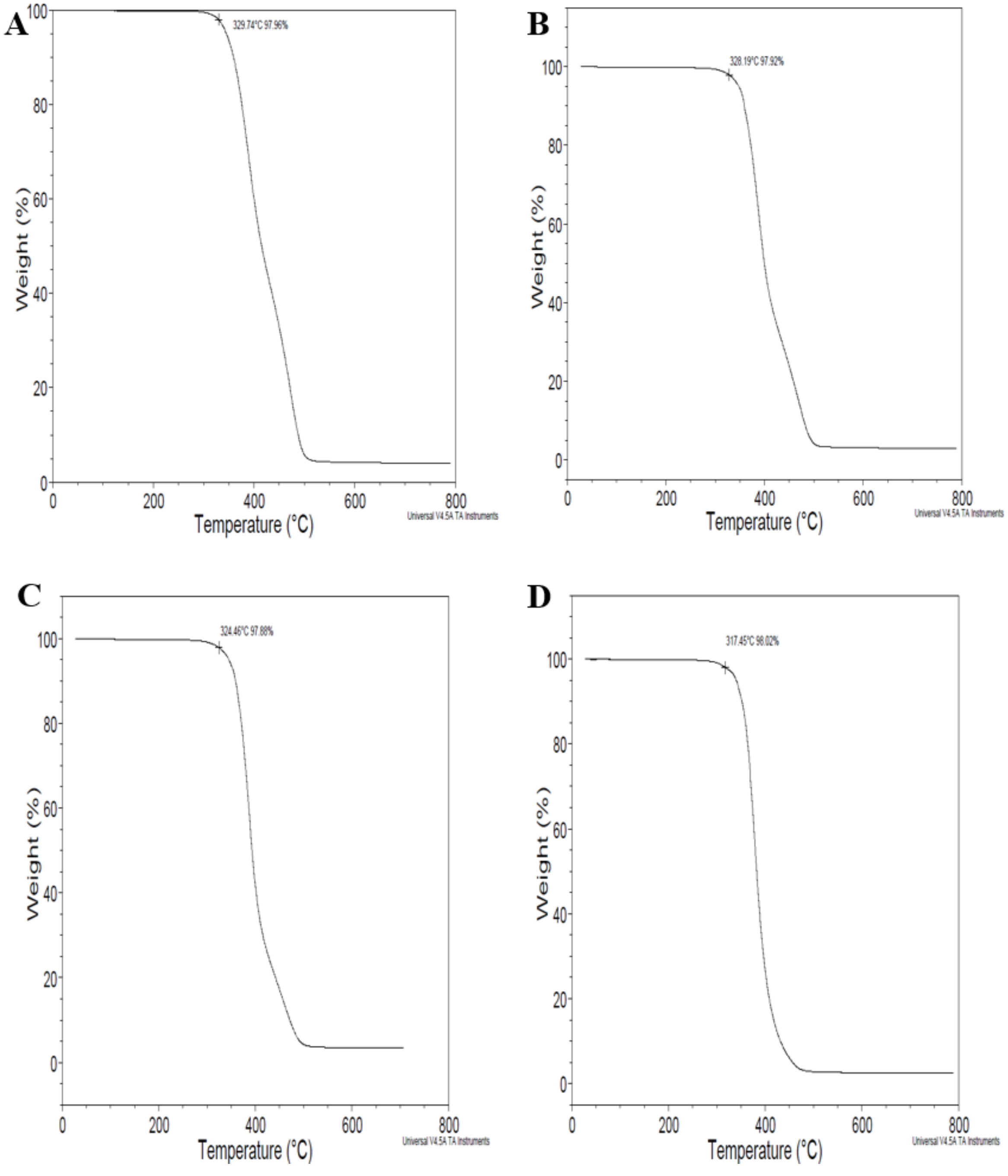

Figure S3: TGA traces for four polyanhydrides to check their decomposition temperatures before doing the DSC experiments. Initial mole ratios of monomers used in samples were PNA: PETMP: EGDT= A) 100:100:0. B) 100:75:25. C) 100:50:50. D) and 100:25:75. The decomposition temperatures were seen at 329 °C, 328 °C, 324 °C, and 317 °C respectively.

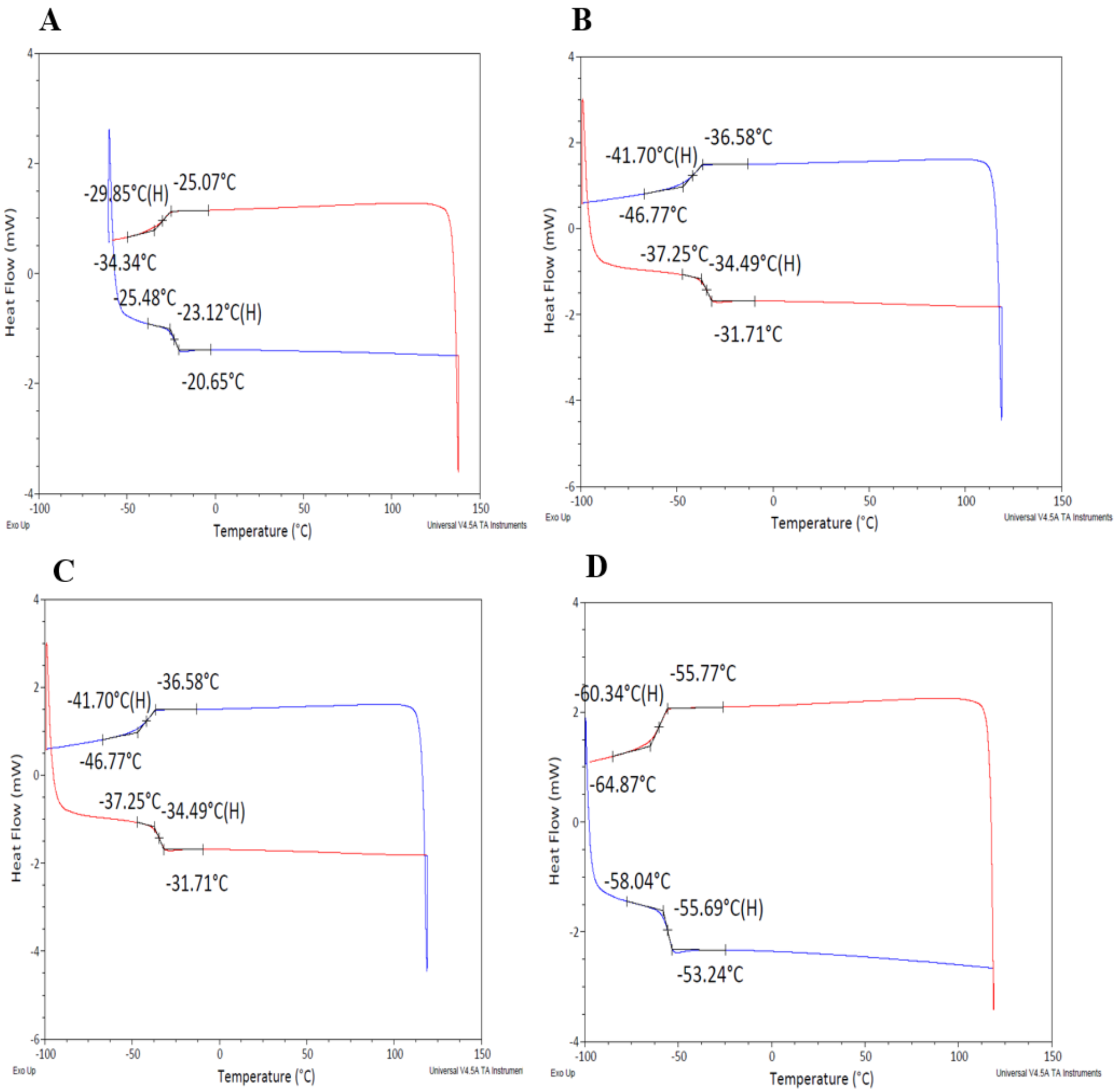

Figure S4: DSC of four different polymers with initial mole ratios of PNA: PETMP: EGDT equal to: A) 100:100:0. B) 100:75:25. C) 100:50:50. D) 100:25:75.Tg were specified from the second cycle of heat/cool steps and -25.1°C, -36.6°C, -48.9°C, and -55.8°C respectively.

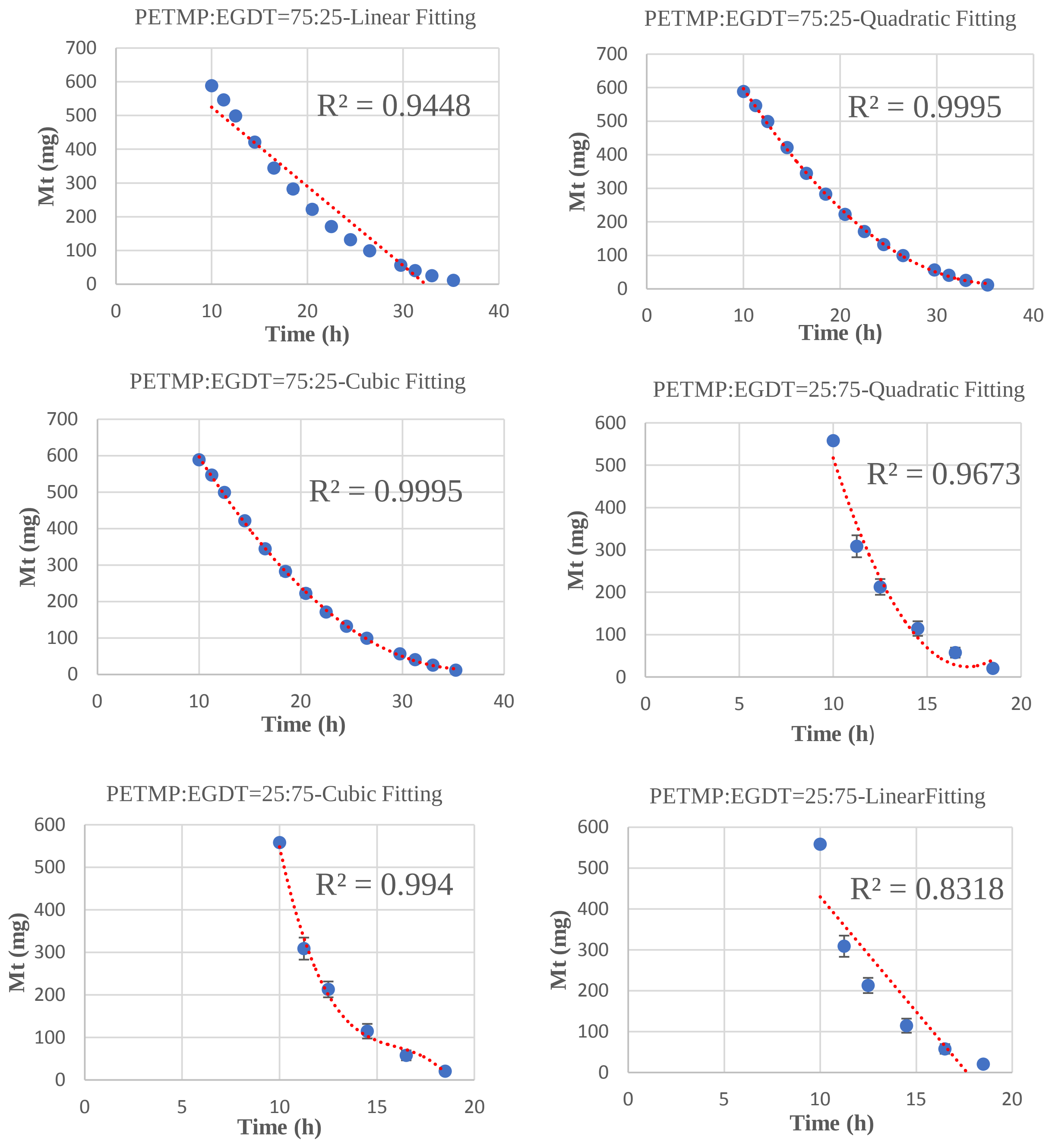

Figure S5: Fitting mass loss data for cylindrical tablets. Linear, quadratic, and cubic fitting curves to the mass loss data of PAHs with initial mole ratios of PETMP: EGDT=75:25 and 25:75. The R^2 values are shown on the graphs.

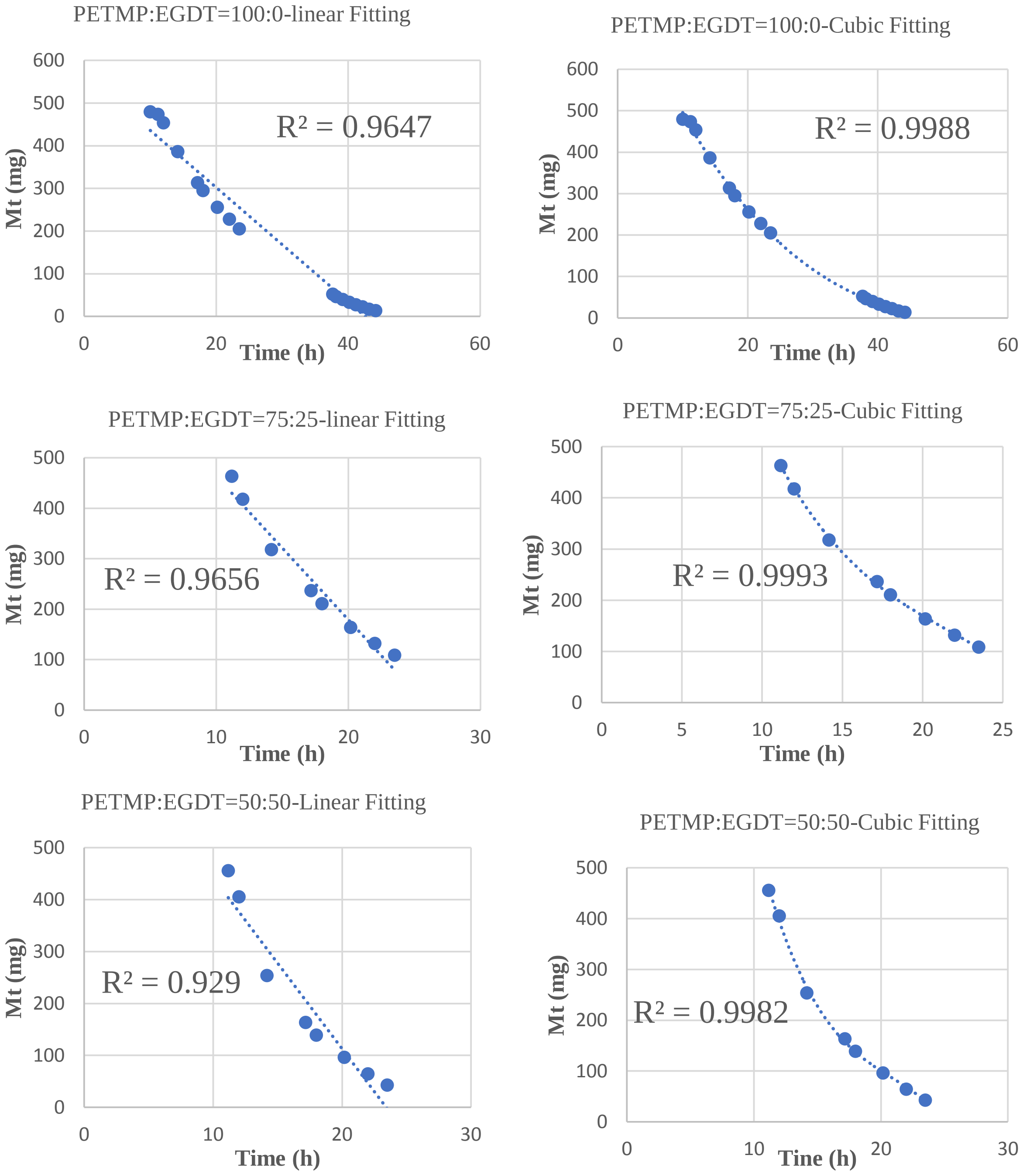

Figure S6: Fitting mass loss data for cuboid polymers. Linear, and cubic fitting curves to the mass loss data of PAHs with initial mole ratios of PETMP: EGDT=100:0, 75:25, and 50:50. The R^2 values are shown on the graphs.

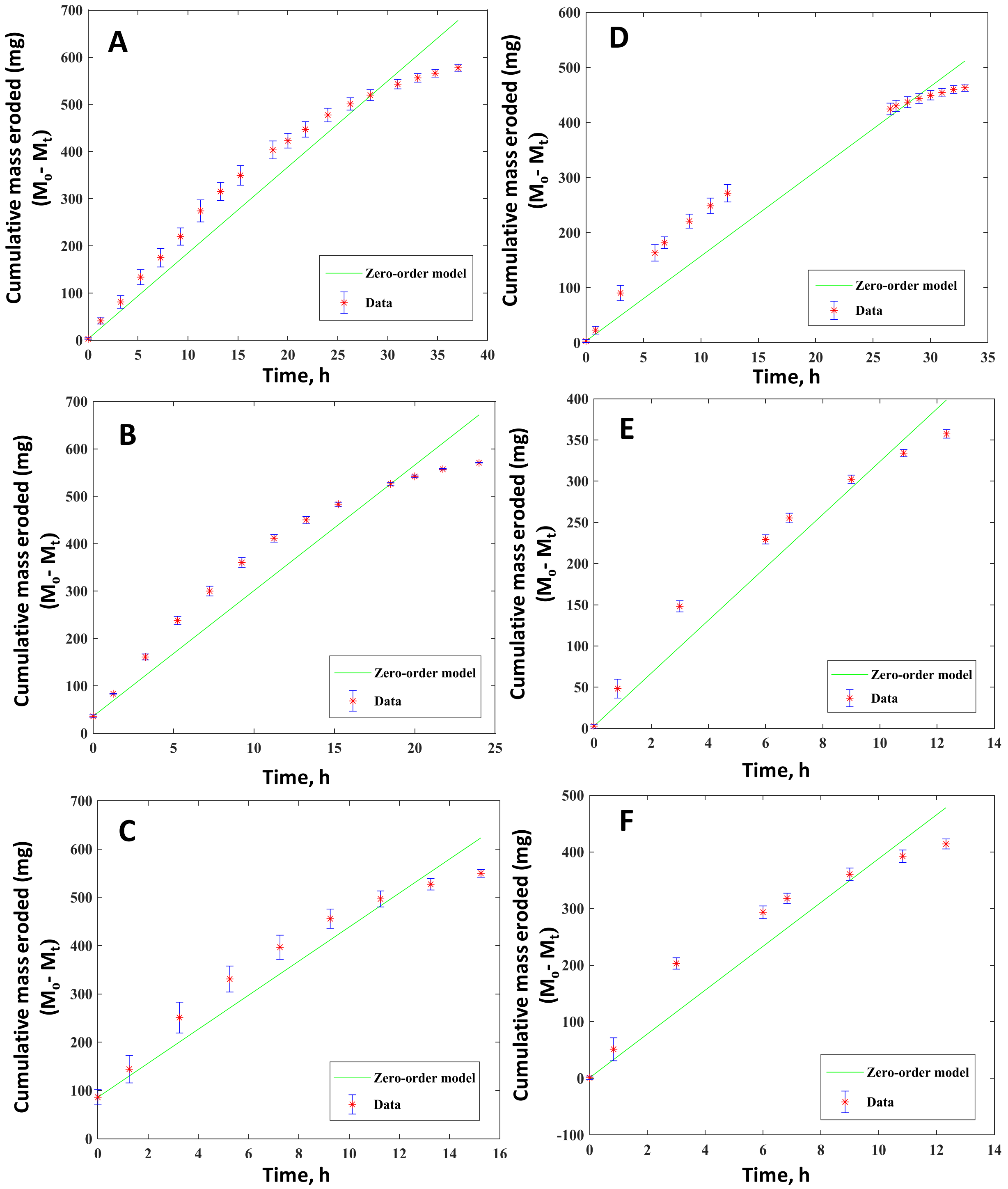

Figure S7: Cumulative mass eroded as a function of time fitted to zero-order kinetic model for cylindrical and cuboid PAH tablets with different crosslinking ratios in PNA:PETMP:EGDT systems. Mole ratios of 100:100:0 (A, D), 100:75:25 (B,E) and 100:50:50 (C,F). Red dots and green lines are experimental data and fitted curves, respectively.

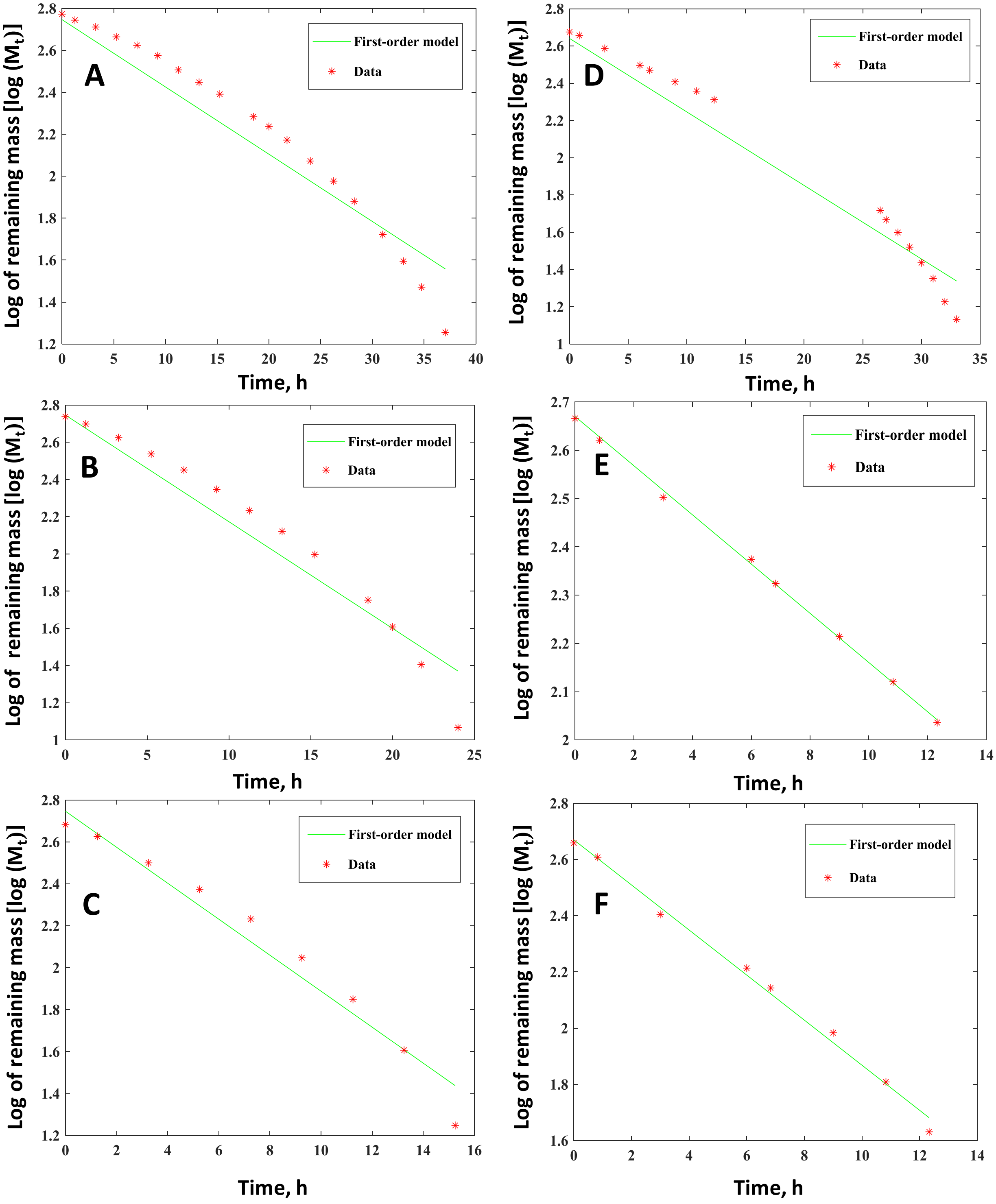

Figure S8: Log of the remaining mass as a function of time fitted to first-order kinetic model for cylindrical and cuboid PAH tablets with different crosslinking ratios in PNA:PETMP:EGDT systems. Mole ratios of 100:100:0 (A, D), 100:75:25 (B,E) and 100:50:50 (C,F). Red dots and green lines are experimental data and fitted curves, respectively.

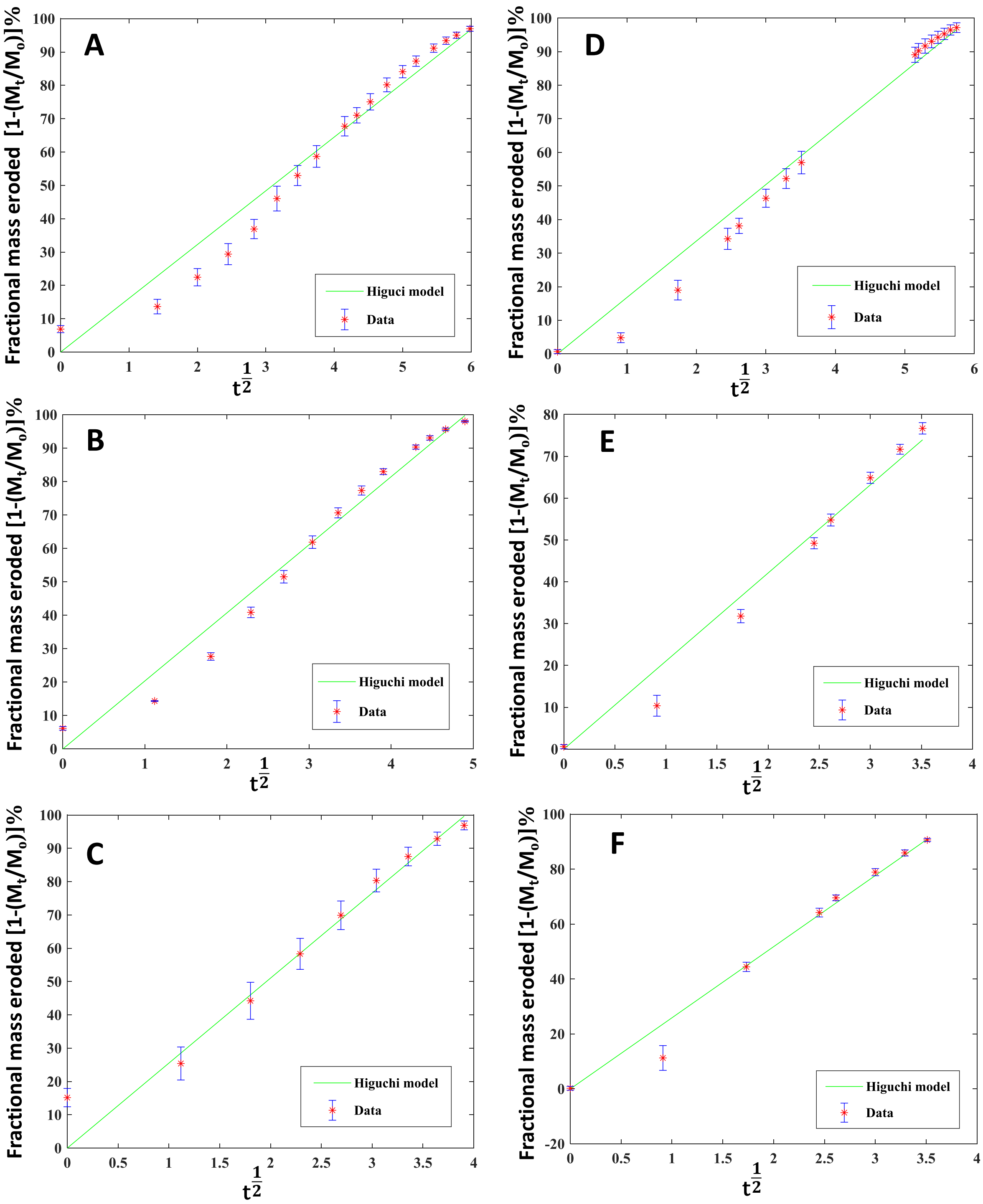

Figure S9: Fractional mass eroded percentage as a function of square root of time fitted to Higuchi kinetic model for cylindrical and cuboid PAH tablets with different crosslinking ratios in PNA:PETMP:EGDT systems. Mole ratios of 100:100:0 (A, D), 100:75:25 (B,E) and 100:50:50 (C,F). Red dots and green lines are experimental data and fitted curves, respectively.

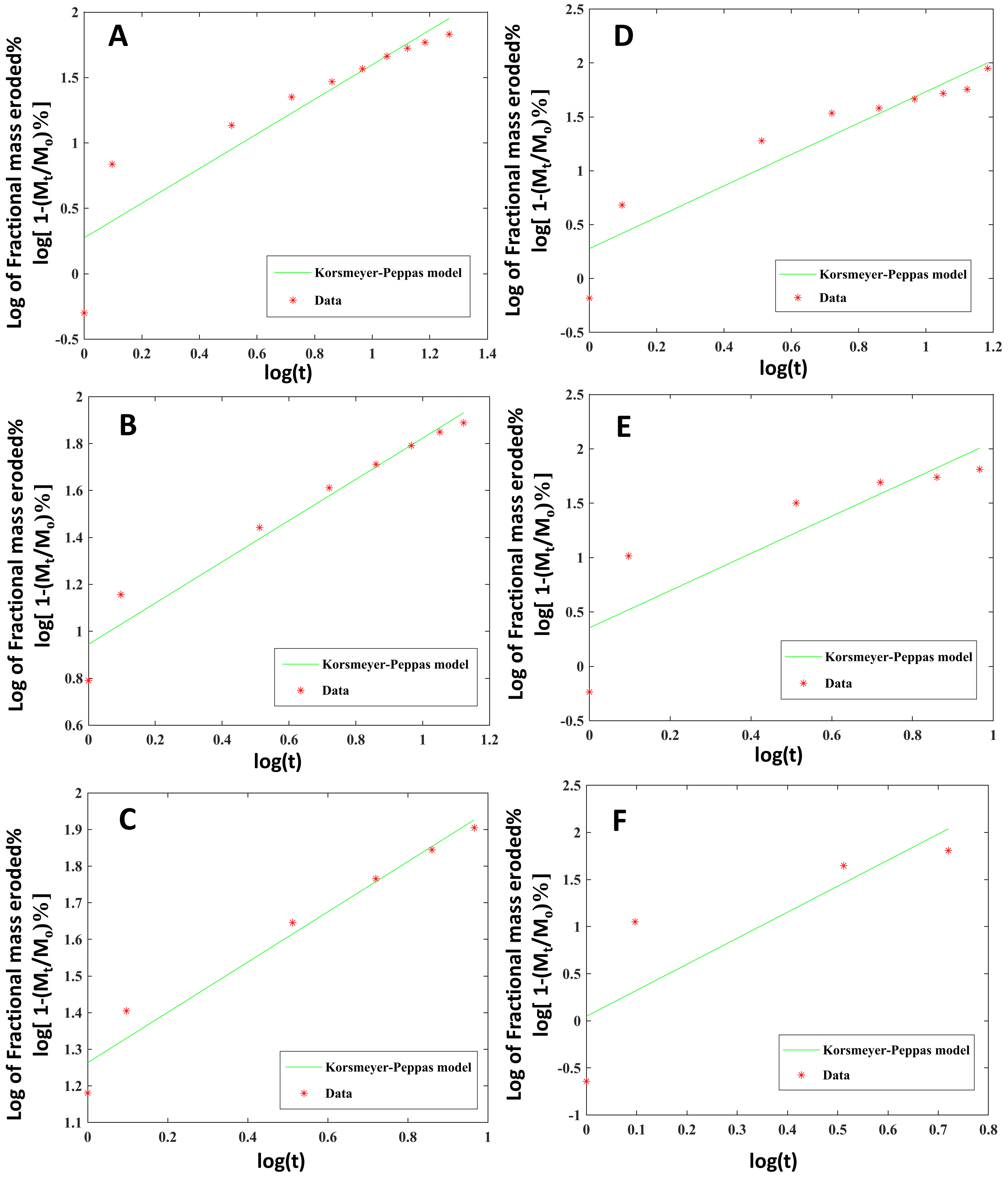

Figure S10: Log of the fractional mass eroded percentage as a function of the log(time) fitted to Korsmeyer-Peppas kinetic model for cylindrical and cuboid PAH tablets with different crosslinking ratios in PNA:PETMP:EGDT systems. Mole ratios of 100:100:0 (A, D), 100:75:25 (B,E) and 100:50:50 (C,F). Red dots and green lines are experimental data and fitted curves, respectively.

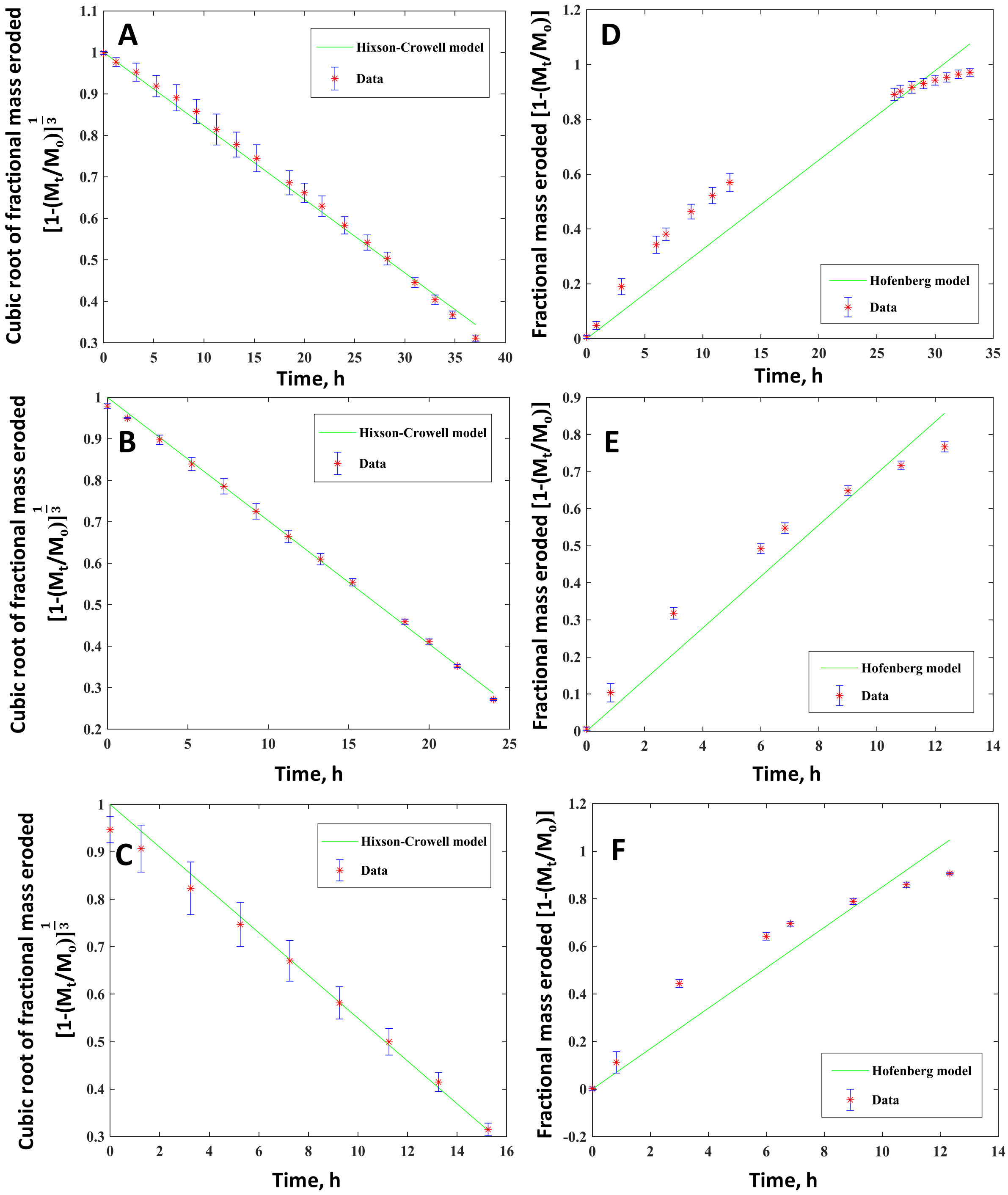

Figure S11: Cubic root of fractional mass eroded as a function of time for cylindrical (A-C) and cuboids (D-F) PAH tablets with different crosslinking ratios in PNA:PETMP:EGDT systems. Mole ratios of 100:100:0 (A, D), 100:75:25 (B,E) and 100:50:50 (C,F). Fitted kinetic models with red dots (experimental data) and green lines are the fitted curves for Hixson-Crowell (A-C) and Hopfenberg (D-F) release kinetic models.
